# Accelerated Reaction Exploration across Scales: A Hybrid Operando and Modeling Study of Oxidation Kinetics in Monolayer Tungsten Disulfide

**DOI:** 10.1021/jacs.6c03511

**Published:** 2026-05-20

**Authors:** Jad Jaafar, Ye Fan, Maryam Kazemzadeh-Atoufi, Ryo Mizuta, Jinfeng Yang, Jack E. N. Swallow, Elizabeth Jones, Matthijs Van Spronsen, Georg Held, Robert S. Weatherup, Peter Voorhees, David J. Wales, Gabor Csanyi, Stephan Hofmann

**Affiliations:** † Department of Engineering, 2152 University of Cambridge, Cambridge CB3 0FA, U.K.; ‡ Department of Materials Science and Engineering, 3270 Northwestern University, Evanston, Illinois 60208, United States; § Department of Materials, 6396 University of Oxford, Oxford OX1 3PH, U.K.; ∥ Department of Chemistry, 5292 University of Manchester, Manchester M13 9PL, U.K.; ⊥ 120796 Diamond Light Source, Didcot OX11 0DE, U.K.; # Department of Chemistry, 2152 University of Cambridge, Cambridge CB2 1EW, U.K.

## Abstract

The commercialization of emerging materials is hindered by the empirical nature of process development, with the underpinning solid-state reaction kinetics remaining elusive due to their inherent multistep and multiscale character and the vast configurational and parameter space. We combine high-throughput *operando* scanning electron microscopy (OSEM) with extended classical continuum and phase-field simulations and atomistic machine-learned interatomic potential (MLIP) surrogate models to demonstrate effective foundational reaction exploration, using thermal oxidation of chemical-vapor-deposited monolayer WS_2_ across a temperature range of 450–680 °C as our benchmark reaction. OSEM provides statistically relevant reaction data sets of spatiotemporal basal plane nucleation kinetics and propagation of tens of thousands of individual 1D reaction facets, revealing time-dependent rates. By extending Avrami theory and employing a calibrated phase-field model, we extract an apparent nucleation barrier of 1.1 eV and show that the complex rate behavior arises from mixed reaction–diffusion control. Atomistic reaction exploration via MLIPs guided by these experimental data reveals that oxidative reaction chains leading to W volatilization and etch pit formation are driven not by the most common sulfur or substitutional oxygen point defects but by more complex defects such as W vacancies. MLIP diffusion barrier screening identifies the role of chemisorbed hydroxyl species for this reaction scenario, while systematic screening of 1D edge configurations and their terminations uncovers the structural origins of the pronounced in-plane reaction anisotropies. We discuss the potential of our synergistic approach to effectively bring experimental and computational approaches closer together and accelerate critically required process discovery for advanced materials.

## Graphical abstract

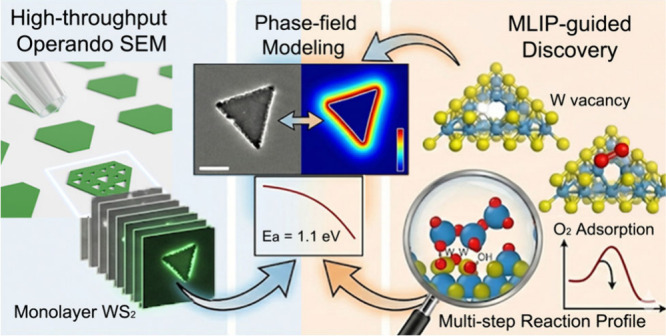

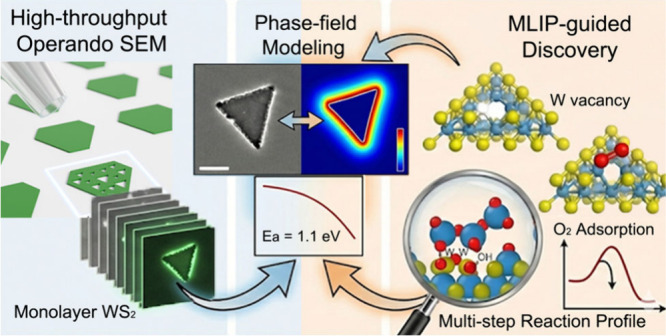

## Introduction

Commercialization of emerging materials relies on process discovery and design. While significant progress has been made in the digitalization and acceleration of materials discovery,^1–3^ materials process development remains largely empirical, dominated by Edisonian trial-and-error methods. Such methods are neither efficient nor able to provide a basis to drive scientific understanding and deeper innovation. The underpinning reactions are inherently multistep and multiscale, connected to a vast configurational and parameter space, which continues to pose a grand challenge to both theoretical and experimental approaches.^4–6^

Oxidation epitomizes this challenge. It is fundamental across the entire material life cycle, from synthesis and processability to environmental and operational stability, lifetime assessment and reuse in applications ranging from catalysis and energy storage to biotechnology and optoelectronics.^7–9^ Layered materials like transition-metal dichalcogenides (TMDs), such as WS_2_, have pushed technology roadmaps to the monolayer level,^10–13^ but foundational reactions like oxidation remain poorly understood,^14–24^ compounded by striking anisotropies in their basal-plane/edge asymmetry and local coordination^25–30^ as well as the significant role of proximity effects and intrinsic/extrinsic disorder.^18,24,31–39^

The key reactions for monolayer TMDs including crystal growth, etching, oxidation and transformations are governed by heterogeneous nucleation and subsequent lateral propagation of 1D reaction fronts. While nucleation can involve atomic point defects, the length scale for propagation can exceed 100 μm. Capturing spatiotemporal reaction kinetics requires *operando* metrology that can bridge such scales, while being compatible with a wide range of technologically relevant substrates and process atmospheres. Many experimental insights into TMD oxidation have been achieved, from substitutional oxygen incorporation into the basal plane and identifying atomic edge configurations, to showing the vast extent of the associated parameter space.^26,31,32,35,40^ However, the trade-offs between required resolution, throughput, and sample/reaction compatibility leave these findings fragmented.^41,42^ Capturing reaction rate data in an effective, statistically relevant fashion remains a key bottleneck that currently precludes effective development of data-driven approaches.

This challenge is compounded by computational limitations.^4^ While first-principles methods, such as density-functional theory (DFT), provide reasonably accurate energies and forces—and, when coupled with geometry optimization, can deliver activation barriers for elementary steps with predefined end states—mapping multistep reactions remains prohibitively time intensive and computationally expensive. Hence, DFT studies have focused on select atomistic details such as the initial O_2_ interaction with the pristine TMD basal plane, single chalcogen vacancies (V_S_), and ideal zigzag edge configurations.^43–46^ Ab-initio molecular dynamics (AIMD) has been used to model aspects of TMD oxidation,^46,47^ but its computational demand necessitates very high temperatures to observe rare events within tractable time scales, meaning these processes may not be kinetically relevant under realistic processing conditions. Kinetic Monte Carlo (KMC), while comparatively computationally inexpensive, is fundamentally constrained by its dependence on knowledge of reaction pathways and associated rate constants, which are often unavailable and expensive to calculate. Mesoscale and macroscale continuum formalisms rely heavily on parameter choices and assumptions, such as constant nucleation or reaction rates in the classical Johnson–Mehl–Avrami–Kolmogorov (JMAK) framework and reaction limited regimes for kinetic Wulff plots.^25,26,40^ For emerging materials like monolayer TMDs such parameters and assumptions remain difficult to establish and verify.

Here, we implement a multiscale strategy that couples high-throughput *operando* scanning electron microscopy (OSEM) with phase-field, atomistic machine-learned interatomic potential (MLIP) surrogate models and characterization of the corresponding energy landscape, to demonstrate the effective capture of thermal oxidation kinetics of monolayer WS_2_ across a temperature range of 450–680 °C. OSEM enables effective screening of the entire reaction progression from nm to mm scale, comprising spatiotemporal basal plane nucleation kinetics and the propagation of tens of thousands of individual 1D reaction facets. The OSEM image and video interpretation is supported by chemical information from correlative *operando* X-ray photoelectron spectroscopy (XPS) and postprocess sample characterization. This allows us to distill statistically relevant experimental rate data sets. We find that both nucleation and edge reaction rates can produce complex time dependences, which challenges widely held assumptions of constant rates at a given temperature and purely reaction limited behavior. Via an extension to Avrami’s classic theory and a calibrated phase-field model, we extrapolate relevant activation energies and effective diffusion lengths and relative interface mobilities. We use this framework to guide an atomistic exploration of the complex energy landscape associated with multistep oxidation and volatilization, employing MLIP based on the MACE architecture.^48,49^ We trained these potentials on >3,000 DFT structures to achieve near-DFT accuracy at a fraction of the computational cost. Employing state-of-the-art exploration of the energy landscape with basin-hopping global optimization using the GMIN code^50–52^ and characterization of transition states and multistep pathways using OPTIM,^53–55^ we efficiently navigate previously intractable reaction sequences involving numerous intermediates. We establish a deeper understanding of multiscale reaction kinetics that connects diverse previous literature and discuss the wider potential of our synergistic approach to accelerate critically required process discovery for advanced materials.

## Results

### *Operando* Micro-Reaction Platform

Our high-throughput reaction screening is based on a high temperature heating stage combined with a robotically actuated quartz micronozzle locally injecting air onto heated WS_2_ domains (Figure 1a, see Methods). Air represents the practically most relevant oxidative atmosphere, and we find that the reaction outcomes described here also apply under other oxidation conditions (see SI Figure S12 for SEM images following O_2_ exposure).Test-particle Monte Carlo (TPMC) simulations, Figure 1b, indicate a local air pressure of ∼6 mbar across a ∼50 μm diameter area for a typical nozzle diameter of 10 μm and height of 50 μm above the sample. The global background pressure in the SEM thereby remains ≤5 × 10^–5^ mbar during dosing, allowing selective local thermal oxidation while imaging the reaction with a high-resolution in-lens secondary electron (SE) detector. The SEM large field of view enables the simultaneous tracing of many (typically ∼10^4^) oxidation features across a WS_2_ domain. We adopt an array-style interrogation approach, where individual WS_2_ domains (hundreds on a typical substrate) are sequentially exposed, resulting in statistically relevant data sets. We developed a software package to semiautomatically correct drift and distortions as well as extract feature dynamics from an image sequence in a full data tree structure (see SI).

**Figure 1:**
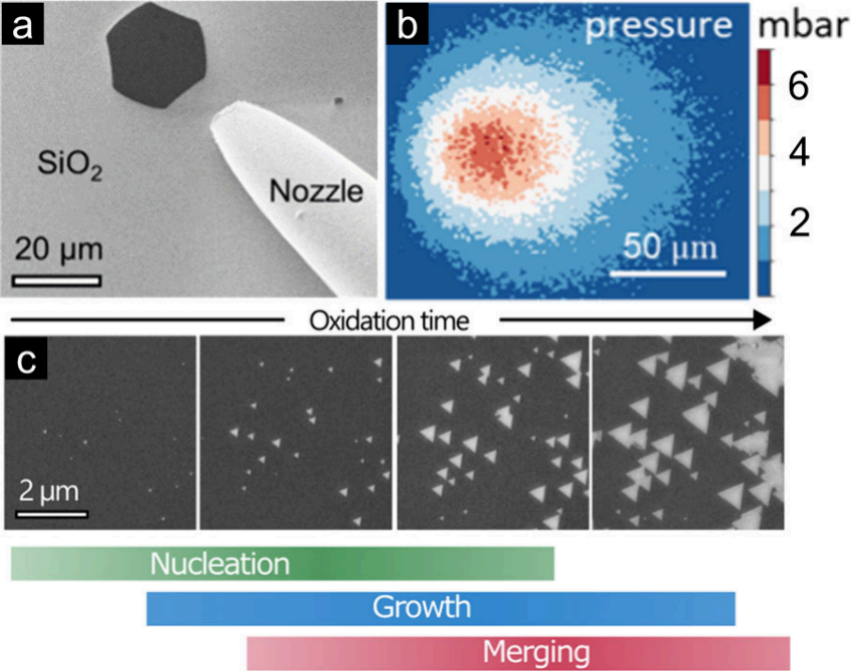
***Operando* microreaction platform.** (a) OSEM image during localized gas injection onto WS_2_ monolayer grown on SiO_2_. The image is taken at 500 °C and a global background pressure of ≤5 × 10^–5^ mbar. (b) TPMC simulation of local pressure underneath a 10 μm diameter nozzle with 50 μm separation above the substrate. (c) OSEM image sequence revealing the spatiotemporal characteristics of the oxidation reaction, focusing on the center of a WS_2_ domain at 680 °C (see SI, video S1, for corresponding video data).

Figure 1c shows a representative spatiotemporal fingerprint of the monolayer WS_2_ oxidation reaction. It manifests itself by discrete nucleation on the basal plane of the WS_2_, resulting in etch pits which subsequently expand and merge via the propagation of well-defined 1D reaction facets. We support the OSEM feature interpretation by correlative *operando* XPS, allowing us to discern also the chemical changes occurring under reaction conditions, in addition to detailed pre- and postprocess sample characterization including optical spectroscopy (Raman, PL; see SI) and atomic force microscopy (AFM; see SI). Figure 2 shows reaction resolved W 4f_7/2_ and 4f_5/2_ XPS core level spectra and extrapolated XPS intensity maps for oxidation at 470 and 600 °C, respectively (see SI for corresponding O 1s and S 2p core level data). XPS survey scans (see SI) show no measurable sample contamination. The chemical shift in the W 4f doublet binding energies (BEs) allows a clear distinction between pristine WS_2_, with a 4+ W oxidation state [W­(IV)], and the resulting W oxide, dominated by a 6+ W oxidation state [WO_3_, W­(VI)]. The gradual, temperature dependent decay of the W­(IV) intensity (Figure 2) is consistent with the loss in pristine WS_2_ basal plane area (Figure 1c). These *operando* XPS results show that the WS_2_ locally converts to WO_3_ and various suboxides, which in turn exhibit a thermally activated sublimation rate. The concurrent decay of the W­(VI) intensity at 600 °C indicates that the sublimation-driven loss of tungsten oxides exceeds their rate of formation. During the oxidation, the W­(IV) doublets do not shift nor broaden. This agrees with the Raman and PL analyses (see SI), confirming that the remaining monolayer WS_2_ retains a high degree of crystallinity. The initial broad benchmarking of the observed SE contrast enables us to transition to a self-consistent, high-throughput OSEM investigation. In the following, we analyze OSEM data sets for each of the three key reaction phases and thereby identify reaction characteristics that we then connect to multiscale modeling to achieve a deeper understanding of the underpinning mechanisms.

**Figure 2:**
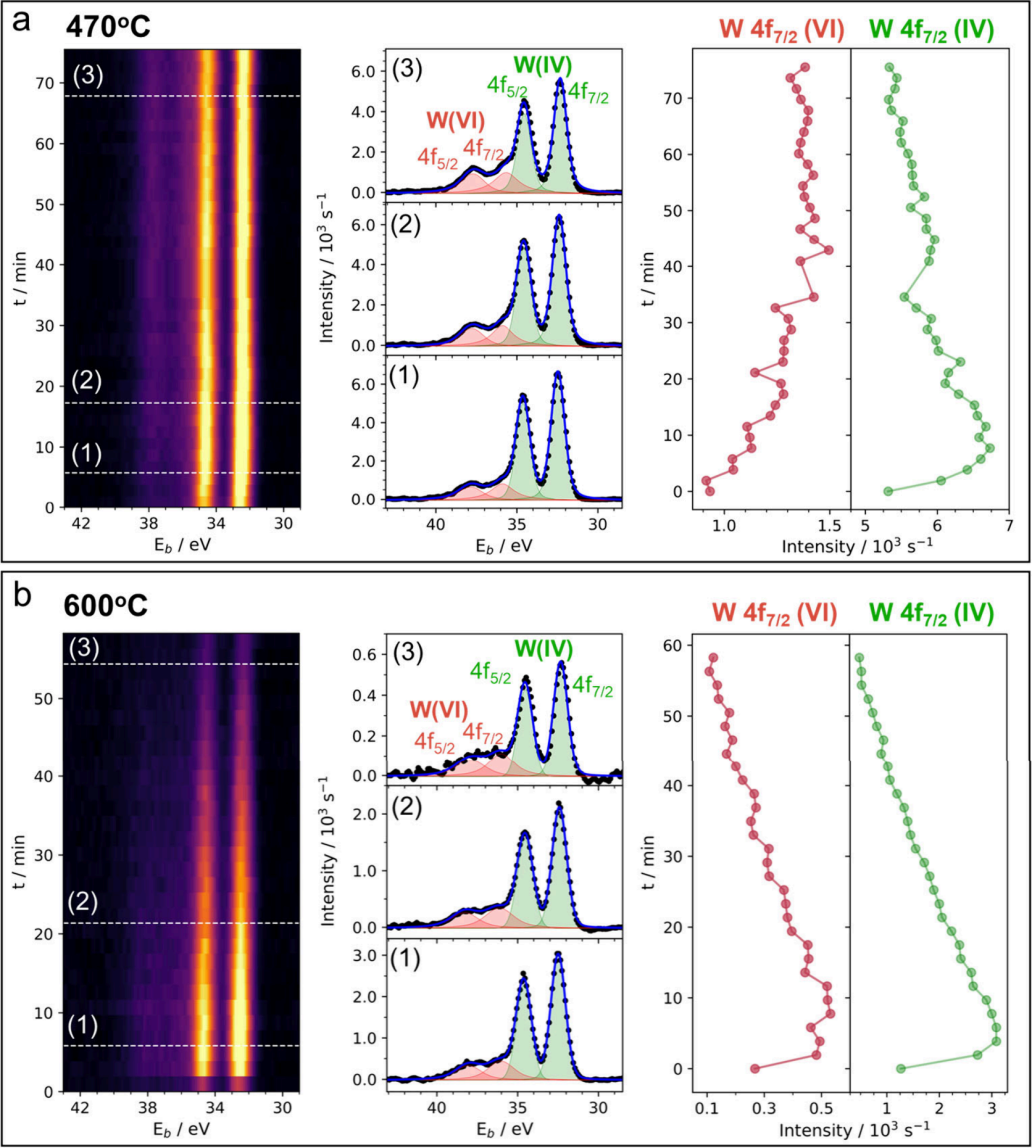
***Operando* XPS of monolayer WS**_**2**_ **oxidation** at ∼0.4 mbar O_2_ at (a) 470 °C and (b) 600 °C. For all spectra, background subtraction has been applied via Shirley fitting. Initially, W 4f_7/2_ and 4f_5/2_ doublet peaks are observed at binding energies (BEs) of 32.4 and 34.6 eV, corresponding to W­(IV) in pristine WS_2_.^56–58^ During the oxidation at 470 °C, the intensity of W­(IV) XPS peaks decreased with exposure time commensurate with the growth of a weaker W 4f doublet at higher BEs of 35.6 and 37.8 eV. In comparison, the W­(IV) core levels decay rapidly at 600 °C, in line with expected higher thermal oxidation rates. The W­(VI) doublet of WO_3_ is also observed, but, in contrast to the case in thermal oxidation at 470 °C, the peak intensity decays during the reaction at 600 °C, which indicates that the rate of WO_3_ loss by sublimation exceeds its rate of formation for these conditions. See SI for additional *operando* XPS data.

### Basal Plane Nucleation Kinetics

Figure 3 summarizes the spatiotemporal nucleation kinetics of the oxidation reaction based on a representative OSEM data set for a single crystal WS_2_ monolayer domain at 450*°*C. Nucleation hereby refers to the emergence of localized features in SE contrast. The monolayer oxidation and etch process starts and evolves from discrete nucleation sites while the surrounding material remains crystalline WS_2_ (see SI). Figure 3a shows that the nucleation events occur over the entire sample and time period investigated. Our *operando* approach enables us to define etch or growth time *t*_*g*_ via the nucleation time *t*_*nuc*_: *t*_*g*_ = *t* – *t*_*nuc*_, where *t* is the experimental exposure time, and *t*_*nuc*_ is the time when the feature first becomes detectable.

**Figure 3:**
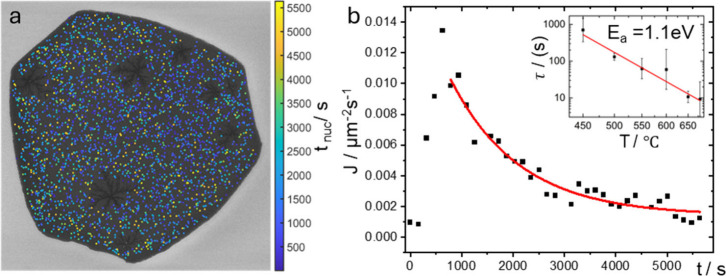
**Nucleation kinetics of oxidation reaction of monolayer WS**_**2**_. (a) Typical spatial distribution of nucleation sites on the basal plane of a single WS_2_ domain as observed by OSEM for oxidation at 450 °C. The color scale represents the nucleation time of every etching pit. Multilayer regions are masked (black areas) and excluded in the statistical analysis. (b) Temporal evolution of the nucleation rate extracted from the same experiment as in (a). The inset shows extrapolated activation energy of characteristic incubation time τ.

Figure 3b plots the nucleation rate, $J=\frac{1}{A}\frac{dN}{dt}$ , where *N* is the accumulated total number of growing nuclei, and *A* is the unetched WS_2_ basal plane area. Note that *A* is time-dependent, due to the reaction dependent loss of basal plane area. Characteristic for all temperatures probed, *J* exhibits a peak-shaped time dependence that converges to a nonzero value for longer *t*. Nucleation is thus not confined to the initial air exposure, a widespread assumption made by ex-situ studies,^25,28^ neither is the nucleation rate a constant, a widespread assumption for modeling such reactions. Classical JMAK theory predicts the change in oxidized area as a function of time. Our *operando* measurements enable us to go beyond measuring the area transformed and examine the nucleation rate of the domains that underpins JMAK theory. We find a nonconstant and time-dependent nucleation rate and thus extend Avrami’s classic theory (see SI) by assuming that nucleation sites are not only activated and consumed during the formation of pits, but also are created:

$$J=\frac{1}{A}\frac{dN}{dt}=\left(\frac{\mathrm{\rho ̅}}{\tau }-k\right)e^{-t/\tau }+k$$ 1

where ρ̅ is the initial density of possible nucleation sites, *k* is the rate of possible new nucleation sites formed during the experiment, *k* > 0, and τ is a characteristic incubation time. If k≪ρ̅/τ , or the rate of increase of possible nucleation sites is slower than the characteristic decay of the initial density, then the nucleation rate *J* decays exponentially to a finite homogeneous nucleation rate as experimentally observed. This time dependence of *J* is not assumed in classical JMAK theory and, thus, JMAK theory is not applicable to these experiments. This allows us to determine the activation energy for the process setting τ (inset of Figure 3b). The extrapolated activation energy of 1.1 eV is the maximum for the activation of any step across the entirety of the reaction chain.

To explain the initial nucleation density and to utilize the 1.1 eV nucleus-to-growth barrier inferred from the nucleation decay rate, we train a MACE ML potential (on thousands of relevant DFT structures) as a DFT-fidelity surrogate for path calculations. We consider oxygen-induced sulfur removal, a key initial step in oxidative etching.^47,59^ For O_2_ as the oxidant, this process comprises (i) the initial oxidant attack to form an OSO–W-like adduct and (ii) the first irreversible S-loss (SO/SO_2_, i.e. S→O exchange).^47^ Nudged elastic band (NEB) calculations suggested approximate barriers ≥2 eV for S-removal on the pristine basal plane and near isolated point defects sulfur vacancy (V_S_) and substitutional oxygen (O_S_) (Figure 4), based on single point DFT calculations for selected configurations. In tungsten vacancy (V_W_) environments, neighboring S is under-coordinated, effectively catalyzing reactive adsorption, stabilizing the oxidant adduct and also lowering the subsequent S-removal barrier into the ∼1.0–1.2 eV window. It is unknown if co-oxidants such as OH* (a mobile species) or O* could facilitate an alternative, lower energy path to adduct formation than reactive O_2_ adsorption. However, the adduct is required for sulfur removal, and this step is either slow or energetically unfavorable except near V_W_-like environments. The removal of sulfur monoxide, resulting in sulfur loss and the formation of O_S_, follows a similar trend, as shown in SI Figure S21. Calculations in Figure 4 were redone with the OPTIM program to resolve the pathways correctly considering additional defects (see Figure S20, SI video S3). Here we employed the discrete path sampling framework,^53,54^ as outlined in the Methods section.

**Figure 4:**
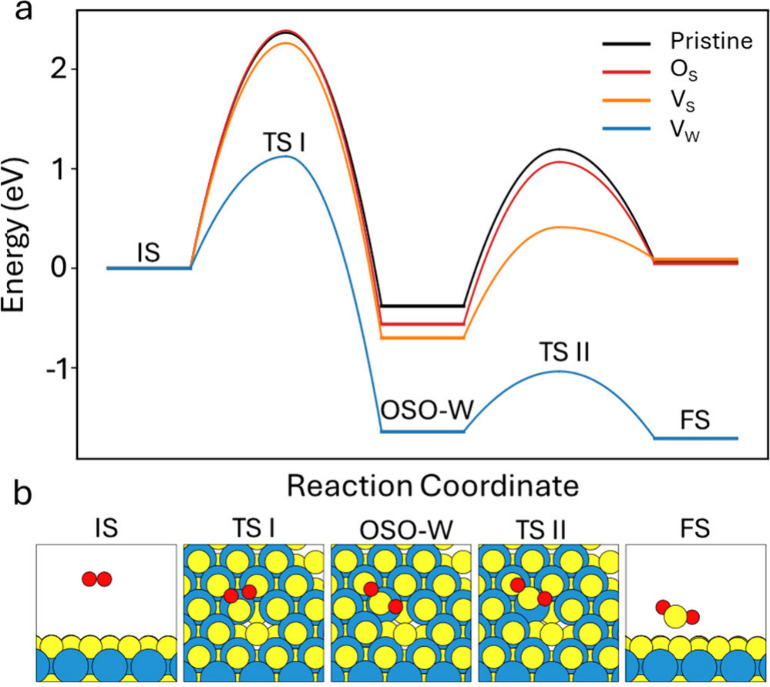
**Reaction profiles for sulfur removal near different defect sites in monolayer WS**_**2**_ **, computed using a MACE potential.** (a) Reaction barriers for pristine, substitutional oxygen (O_S_), sulfur vacancy (V_S_), and tungsten vacancy (V_W_) sites (colored curves as labeled). Each curve represents the DFT single-point energy evaluated along the MACE-optimized nudged elastic band (NEB) approximation of the path for oxygen-assisted desulfurization leading to SO_2_ formation. (b) Representative atomic configurations along the reaction coordinate (NEB configuration pathway) near V_W_. Blue, yellow, and red spheres denote W, S, and O atoms, respectively. The sequence shows the initial state (IS) with adsorbed O_2_, the first transition state (TS I) corresponding to reactive adsorption and O–S bond formation, the OSO–W intermediate characteristic of all sites, the second transition state (TS II) preceding SO_2_ release, and the final state (FS) after sulfur removal. Side views are shown for the initial and final configurations.

To test whether a V_W_ site can propagate beyond its immediate neighborhood, we explore oxidation after the exposed S atoms have been substituted with oxygen, following the structures suggested by the pathways in Figure 4, which suggest a V_S_ that can be passivated by oxygen. Basin-hopping global optimization was used to identify configurations I and III (Figure 5) as possible motifs with added O_2_.^51^ A molecular dynamics run starting from configuration III recovered a nonunique trajectory that involves short-range W–O migrations/reconstructions, revealing new local zigzag (ZZ)-like edge segments exposed by the displacement of a WO_*x*_ motif out of plane (structure X, Figure 5). We then used discrete path sampling in OPTIM to characterize multistep paths connecting configuration I to X. Figure 5 shows that a V_W_ can expand its reactive perimeter with a reaction path for which all barriers up to W oxide sublimation are below 1.1 eV. The experimentally observed nucleation kinetics for the given CVD WS_2_ samples can thus be rationalized by the initial presence of V_W_-type defects.

**Figure 5:**
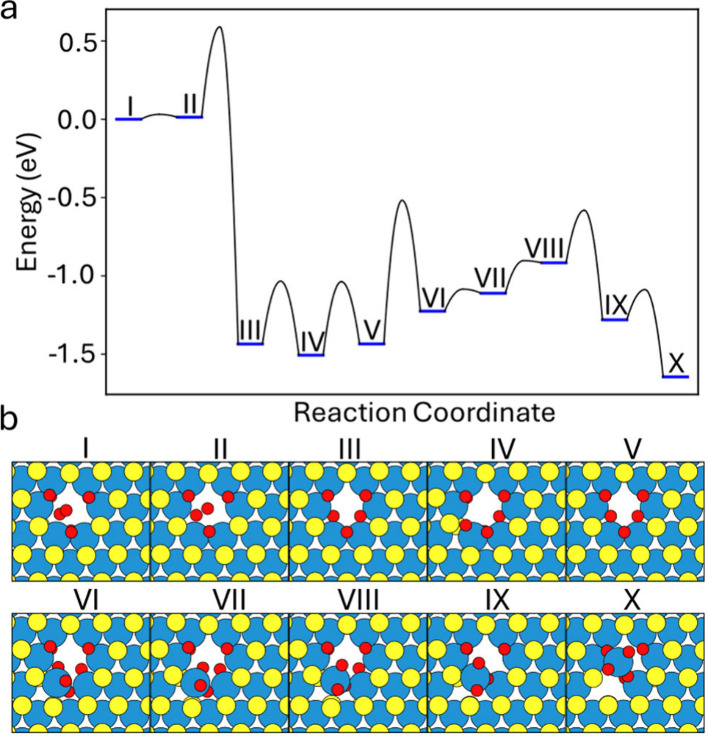
**Reaction profile for the incorporation of O**_**2**_ **into an oxidized V**_**W**_ **defect (postsulfur substitution), computed by using MACE within the discrete path sampling framework using OPTIM**. (a) Energy profile beginning from oxygen attachment to a hollow site (structure I), leading to dissociation and culminating via a series of reconstructions and migrations in the displacement of the WO motif (structure X) and re-exposure of undercoordinated sulfurs (S-ZZ edge orientation). (b) Representative atomic configurations along the reaction coordinate. Blue, yellow, and red spheres denote W, S, and O atoms, respectively. The horizontal axis corresponds to a scaled integrated path length.

### Growth Kinetics of Individual Etch Pits

Following nucleation, our OSEM data show that individual etch pits maintain a strict equilateral triangle shape and are all aligned with respect to each other on a given WS_2_ basal plane (Figures 1c and S1,2). This is consistent with the 3-fold crystal symmetry of 1H WS_2_ and the kinetic selection of 1D reaction facets, whereby the facet with the slowest etch rate dictates the pit shape. This configuration has previously been identified as the metal ZZ facet under oxidative conditions for MoS_2_,^34^ but irrespective of atomistic facet assignment, the well-defined 2D pit shapes allow us here to extract characteristic 1D etch rates based on the areal expansion of the etch pits (Figure 6).

**Figure 6:**
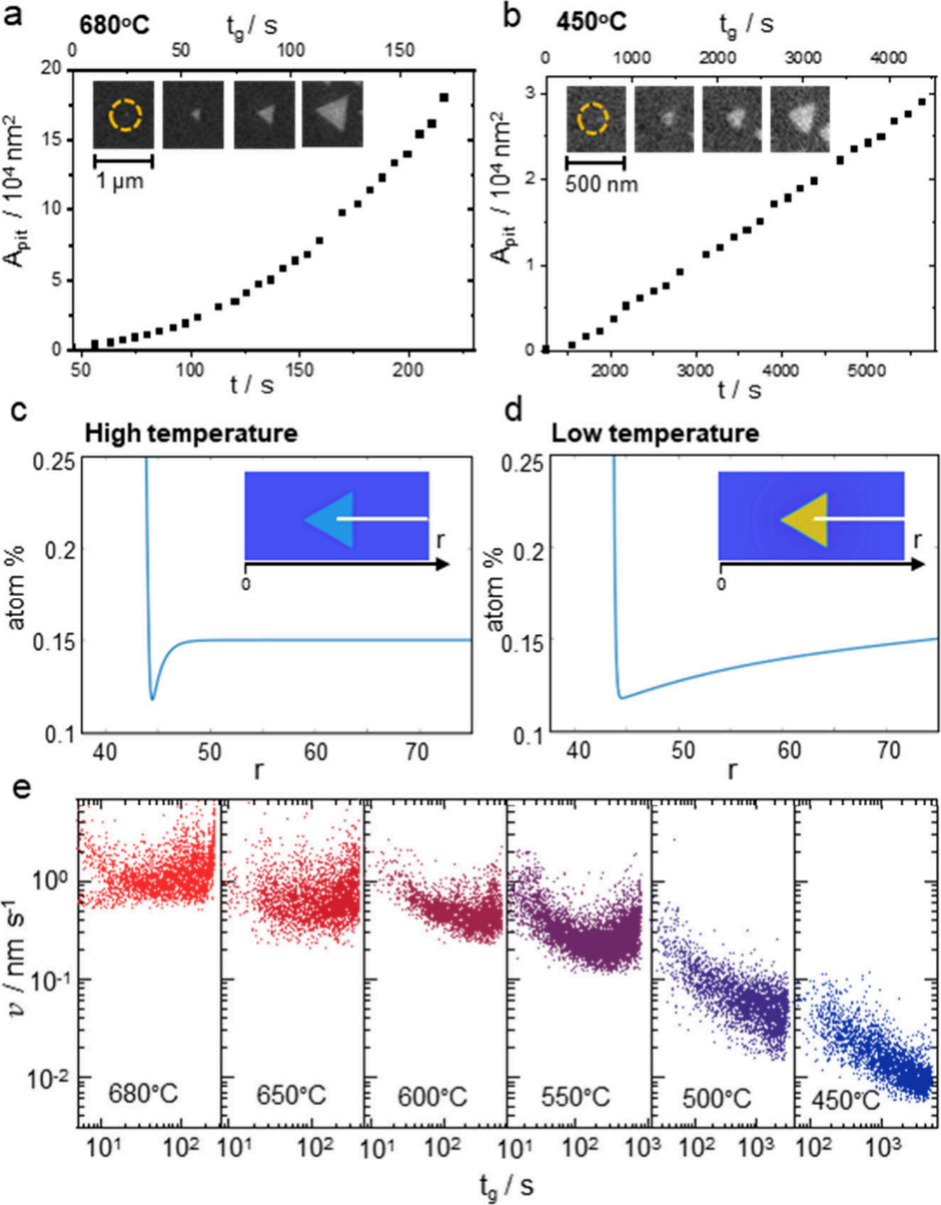
**Oxidation rate of individual pits in monolayer WS**_**2**_. Area of an etch pit, *A_pit_* (*t*), as a function of time at (a) 680 °C and (b) 450 °C. The respective insets of time-resolved OSEM images show the nucleation (orange dashed circles) and subsequent growth of a typical etch pit at the given temperature. Bottom *x*-axis (*t*) is the experiment time; top *x*-axis (*t_g_*) is growth time, where nucleation defines *t_g_* = 0 for a given etch pit. (c,d) Nondimensional oxygen concentration profiles at the WS_2_-oxide interface from the phase field simulations, parametrized for high (c) and low (d) temperatures. Insets show the 2D simulation results from which the 1D concentration profiles are extracted, shown by the white line. (e) Time dependence of the velocity of the facets, *v*, of >10^3^ etch pits for different temperatures.

We find distinct quadratic and linear time-dependencies of the pit area evolution, *A_pit_* (*t*), at 680 and 450 °C, respectively (Figure 6a,b). This corresponds to a constant facet reaction rate (i.e., facet velocity) at high temperature and a time-dependent decrease in reaction rate at lower temperatures. The decrease in reaction rate is most significant at the early stages of reaction, with the median reaction rate decreasing from 0.05 to 0.008 nm/s within about 1000s after nucleation at 450 °C (Figure 6e). In contrast, at 680 °C the reaction rate remains around 1 nm/s, while at intermediate temperatures, Figure 6e, the facet reaction rate or facet velocity tends to initially decrease then stabilize. The stabilized facet velocity increases 2 orders of magnitude from 450 to 680 °C, evidencing the level of thermal activation.

To rationalize these mesoscale facet growth or reaction kinetics, we implement a binary, multiphase order parameter phase field model with three phases (WS_2_, WO_*x*_, gas) in 2D, based on work by Moelans (see SI).^60^ The importance of species impingement relative to the surface diffusion of species is thereby expressed by the interaction length $\xi =\sqrt{D/Q}$ , where *D* is the surface diffusion coefficient of species on top of the WS_2_ monolayer, and *Q* describes the rate of arrival of species from the localized injection of air. When ξ is small relative to the size of the etch pit, i.e. ξ ≪ *h*, where *h* is the apothem, the pit area grows quadratically with time (Figure 6a), meaning that the etch pit edge has a sufficient supply of oxygen and has a constant edge velocity, consistent with facet reaction kinetics controlled dynamics. The corresponding simulated composition profile of the diffusing species has a short-range diffusion field at the WS_2_-pit interface (Figure 6c), consistent with a facet velocity that is limited by the reaction rate at the facet. In contrast, when ξ ≫ *h*, the etch pit area is linearly dependent on time (Figure 6b), resulting in an edge velocity that decays with time. In this case, the rate limiting factor is diffusion along the WS_2_ surface to the etch pit edge. The corresponding composition profile of the diffusing species exhibits a long diffusion field at the WS_2_-pit interface (Figure 6d), consistent with diffusion-controlled kinetics. Our results show that a mixed regime between basal plane diffusion and edge reaction-limited behavior results in a time-dependent reaction rate, and that 2D oxidation is not solely controlled by the chemical reaction at the edges. Classical JMAK descriptions of pit growth are only valid when the velocity of a facet is set by the reaction rate at the facet.

To gain insight into the nature of WS_2_ surface species, we computed diffusion barriers (*E_D_*) for basic candidates (O*, OH*, V_S_, V_W_) using the MACE model trained on DFT (see Methods, Figure S22). Figure 6 indicates diffusion length scales that are on the order of the etch pit size, i.e. 100–1000 nm based on our experimental data. Using the computed barriers, *E_D_*, we estimated diffusion coefficients D, as outlined in the methods section (see Table S1 in Supporting Information). We find that O*, V_S_ and V_W_, with *E_D_* > 2 eV, are not consistent with the phase-field calculated extent of diffusion length. Our computed barriers for O* and V_S_ diffusion agree with other studies.^61–63^ For the given species, adsorbed OH was the only candidate with a consistently lower *E_D_* ≈ 0.4 eV, similar to its value on MoS_2_.^64^

### Pit Merging and Facet Dependent Reaction Rate Anisotropies

Upon further expansion, individual etch pits start to merge. Figure 7a shows a typical merging event at 680 °C (see SI, video S1). While individual etch pits before and after merging show triangular reaction habits, during the merger a range of different facets are exposed. The merger events thus enable direct access to survey reaction facet anisotropy. We capture this anisotropy in the phase field model via the kinetic coefficient, a parameter related to the interface mobility or reaction rate, Figure 7b. Short-range diffusion fields are thereby present at the interfaces between the etch pits and monolayer WS_2_, indicating that diffusion kinetics contribute to the dynamics even at high temperature. The model output is in excellent agreement with the experimental evolution, including the salient region with highly reactive facets and recovery of the crystal habit by the final frame, Figure 7a,b.

**Figure 7:**
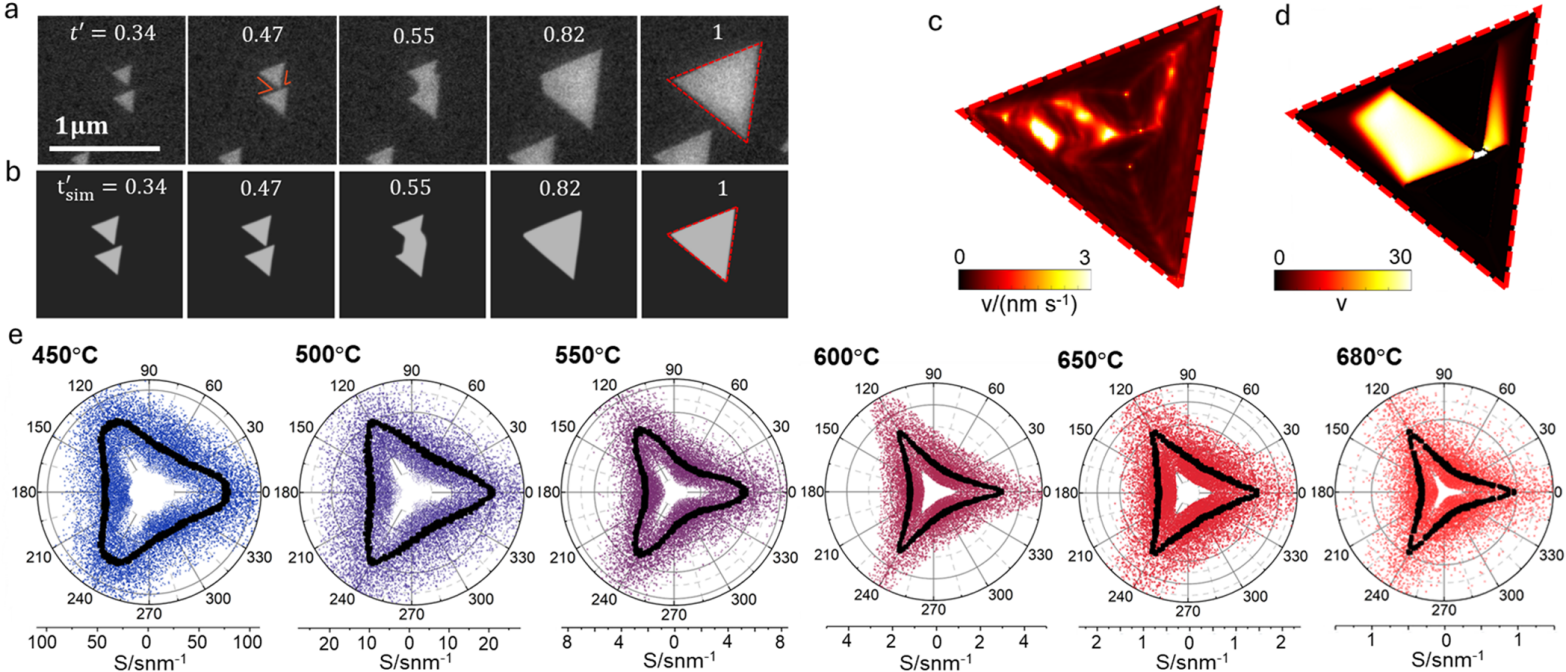
**Anisotropic etch rate.** (a) OSEM images showing the merging of two etching pits at 680 °C. The experiment time normalized by the time of the final frame, *t*′, is labeled on the top for each snapshot. The red polyline in the second frame highlights the salient region formed at the beginning of the merger. (b) Images from phase field merging simulation corresponding to frames in (a). The simulation time normalized by the time of the final frame, *t*_*sim*_^′^, is labeled on the top for each snapshot. (c) Map of the experimental edge moving rate (left panel), from data presented in (a). The three equal tungsten zigzag (W-ZZ) directions are set to 0°, 120°, and 240°, respectively. (d) Map of the edge moving rate in nondimensional units from merging simulation (right panel). (e) Polar plots of the anisotropic reaction slowness (inverse of the edge moving rate) at different temperatures. The slowest reaction direction (W-ZZ direction) is assigned as the 0° (or 120°, 240°), same as the convention in (c). The solid lines overlapping each plot represent the median edge velocity for each direction.

In order to directly measure interfacial velocity of high-reactive edge facets, we adapt the method first proposed by Frank and widely adapted in theories of crystal growth.^65^ A transition time, *T*, map, which tracks the time when the edge of the etch pit arrives at each pixel, was measured from the OSEM image series. From this a reaction slowness, *s*, can be extracted as

s=∇T·n 2

where *T* is the transition time map, the 2D array that gives the time at which the etch interface (i.e., etch pit edge) crosses a given pixel in the experimental frame, and ***n*** is the normal to the facet. The magnitude of the reaction slowness is inversely proportional to the edge local normal reaction velocity *v*. Figure 7c,d plot the observed and modeled reactions rates. The maps show that other 1D WS_2_ facets exhibit significantly higher reaction velocities compared to the slowest reacting edge. The accelerated dynamics during merger can be attributed to the mixed-regime diffusion and reaction-controlled dynamics, where there is a short-range diffusion field with a nonequilibrium composition at the interface. Further contributions include exposed nonequilibrium orientations with larger facet mobilities and negative curvatures which emerge during coalescence.

Figure 7e summarizes the anisotropic reaction slowness along different directions in a format that resembles kinetic Wulff plots, with the difference that all values are directly measured experimentally . The reaction slowness exhibits clear 3-fold symmetry. Increasing the temperature from 450 to 680 °C increases the reaction rate for all 1D crystal edges. The median rates for the slowest and fastest reaction edges at 450 °C are of the order of 80 s/nm and 40 s/nm, respectively, compared to 1 s/nm and 0.3 s/nm at 680 °C. We observe a consistent trend of the ratio between the highest and lowest rates increasing with temperature. Consequently, the shape anisotropy becomes more pronounced at higher temperature.

In WS_2_, the slower propagating facet or edge is typically assigned to one of the two distinct zigzag terminations: the sulfur-terminated (S-ZZ) or the tungsten-terminated (W-ZZ) edge. These edges differ fundamentally in their coordination environments and their electronic structures. Furthermore, under experimental conditions, these edges are not static; they exhibit varying degrees of sulfur coverage (and oxidation), as determined by the local chemical potentials and growth history.

To decouple these factors, we investigated the oxidation kinetics of both S-ZZ and W-ZZ edges across a full range of sulfur coverages (0–100%). The potential energy landscape for oxidation is complex, featuring a multitude of oxygen chemisorption sites and subsequent surface reconstructions. We employed our MACE model within the OPTIM package to perform accurate transition state searches, systematically exploring reaction pathways that connect various pairs of initial and final states. In our analysis of the multistep trajectories, we define the effective activation energy (*E*_*a*_) as the highest barrier encountered en route to an oxidation event, typically characterized by the formation of stable SO_*X*_/WO_*X*_ species. While we tracked trajectories to the deepest identified minima where appropriate, we selectively truncated paths if reaching a more stable state required subsequent reconstructions with barriers comparable to the oxidation step itself. This approach ensures that *E*_*a*_ and Δ*E* reflect the primary kinetic bottleneck and the associated driving force, rather than secondary structural relaxations. Furthermore, while OPTIM naturally identifies various local minima, we did not perform an exhaustive global optimization of the oxidized surfaces (e.g., via basin-hopping), as our focus remained on the initial kinetic accessibility of the edge. The resulting energetics provide a robust map of kinetic accessibility for edge oxidation, as plotted in Figure 8a.

**Figure 8:**
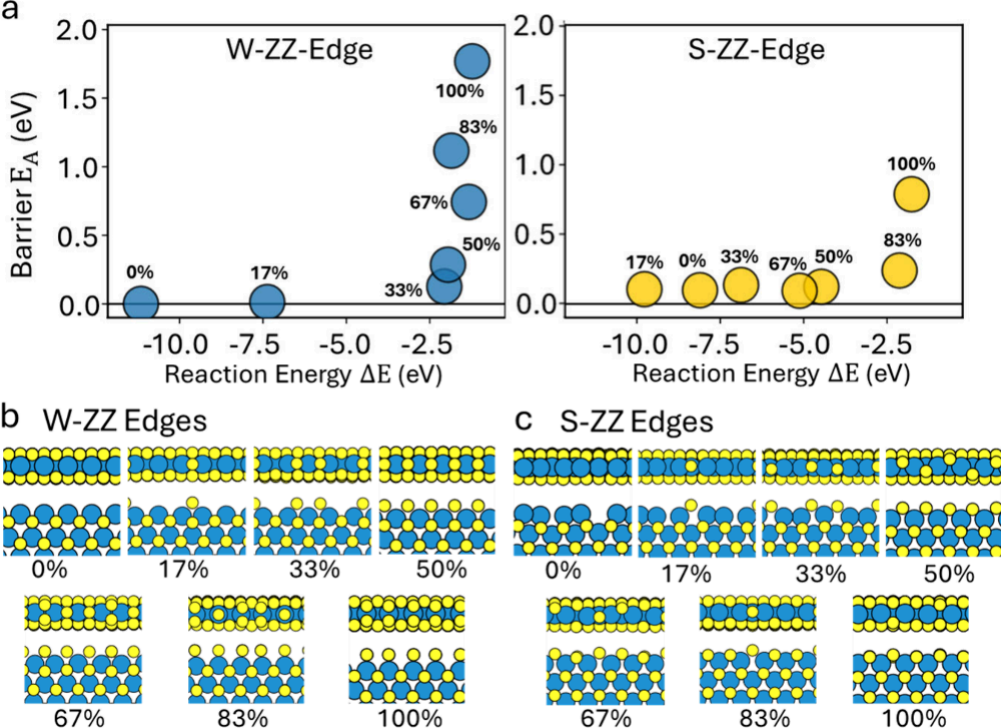
**W-ZZ and S-ZZ edge oxidation reaction energetics computed by using MACE within the discrete path sampling framework implemented in OPTIM.** (a) Plot of the reaction barrier (*E*_*a*_) against the reaction energy (*Δ*E**) for the kinetically dominant pathways identified on S-edges (yellow) and W-edges (blue) for the reactive adsorption of O_2_. Data points are labeled by the corresponding sulfur coverages (in %). The S-edge maintains consistently low barriers across most coverages, while the W-edge exhibits a steep rise in barriers—indicative of passivation—as sulfur coverage increases. Representative structural snapshots of (b) W-ZZ edges and (c) S-ZZ edges featuring two different views per coverage (as annotated below). Structures shown here were optimized using the trained MACE model. Blue and yellow spheres denote W and S atoms, respectively. Full reaction pathways are available in the SI (see Figures S23, S24).

The computed pathways (Figure 8a) reveal a stark contrast in the oxidative stability of the two edges. The S-ZZ edges exhibit more favorable kinetic landscapes: reactions are characterized by large thermodynamic driving forces (highly exothermic Δ*E*) and consistently low barriers (*E*_*a*_ < 0.5 eV) across nearly all coverages. This observation suggests that the S-ZZ edge remains chemically active and prone to rapid recession, regardless of the local sulfur environment. In contrast, the W-ZZ edge displays a strong dependence on sulfur coverage. At low coverages (<67%), the exposed metal centers facilitate low-barrier oxidation. However, as coverage increases, we observe a rapid onset of kinetic passivation. The 100% covered W-edge, which represents the pristine edge exposed during the expansion of a pit into the bulk lattice, exhibits a prohibitive barrier of around 1.77 eV. This divergence explains the anisotropic etching rates observed experimentally. As an etch pit expands into the pristine material, it continuously exposes edges with high sulfur coordination. The S-edge sustains rapid oxidation relative to the W-ZZ edge. Consequently, the propagation of the W-edge likely requires pre-existing sulfur vacancies or a secondary mechanism to strip passivating sulfur atoms, making it the rate-limiting (and thus shape-defining) front of the etching process.

We also explored kinetically relevant atomistic pathways for the oxidative etching of both S-ZZ edges (Figure 9a) and W-ZZ edges (Figure 9b), starting from configurations in which the terminating sulfur atoms are replaced with oxygen (100% coverage). We note that several of the paths related to Figure 8 are kinetically viable pathways that lead to the S–O exchange of the edge-terminating atoms. We chose to find connected pathways starting from these points, with the focus on understanding how etching may be induced.

**Figure 9:**
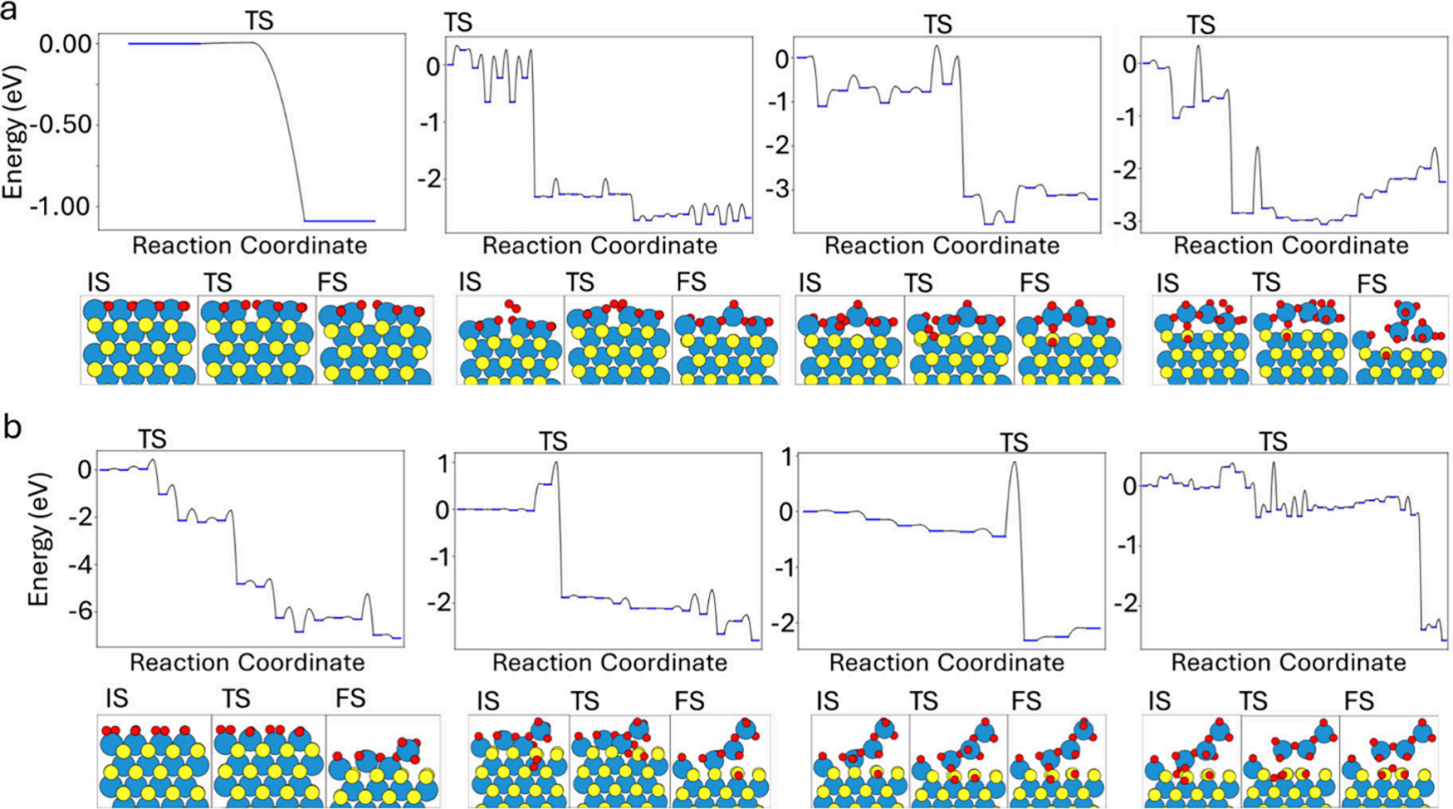
**Multistep reaction profile for the sublimation of tungsten oxide from model WS**_**2**_ **zigzag edges computed for MACE in the discrete path sampling framework using OPTIM to characterize complex pathways**: (a) Sublimation of tungsten oxide starting from an oxygen-terminated sulfur zigzag (S-ZZ) edge. (b) A computed pathway starting from an oxygen terminated tungsten zigzag (W-ZZ) edge. Both paths involve successive reaction with oxygen molecules and reconstructions which culminate in the detachment of WO_*x*_ motifs. Blue, yellow, and red spheres denote W, S, and O atoms, respectively. In sequence (b), an equivalent O_2_ reactive adsorption on the rear S atom, opposite the one oxidized in step 2, precedes step 2 but is not shown here for conciseness. The horizontal axes correspond to scaled integrated path lengths. SI videos S4 and S5 provide movies of the pathways shown here.

Our methodology proceeded iteratively: first, basin-hopping global optimization (via GMIN) identified low-energy reconstructed minima. Second, we modeled the reactive adsorption of O_2_ at under-coordinated sites within these minima. Third, we connected these states using accurate transition state searches (using discrete path sampling and OPTIM as described in the Methods). Pathway construction also involved procedures in the GMIN and OPTIM programs to apply external fields for pulling atoms and catching dissociated species (see Methods). This cycle was repeated until the formation of volatile WO_*x*_ species was observed, successfully yielding complete reaction profiles (Figure 9) spanning tens of intermediates and transition states, all with barriers below 1.4 eV for the elementary steps. The mechanism of volatile W–O_*x*_ release before extensive oxidation occurs is consistent with our XPS data (Figure 2). The similarity in barriers involved in the etching increases the likelihood that the disparity in anisotropy between the two edges can be primarily attributed to the slower initial desulfurization of the W-ZZ edge. The energy landscape supports multiple parallel paths and distinct relaxation time scales, which are not evident from the simple stepwise paths summarized in Figures 5 and 9. Further analysis of the global kinetics will be considered in future work.

## Discussion

OSEM allows us to capture direct, statistically relevant data sets on what constitutes the reaction progression from nm to mm scale, and we achieve synergy in workflow via extended classical continuum and phase-field modeling to interpret data and the use of MLIP to screen and connect to complex, multistep reaction scenarios at atomic scale. We fit the experimentally observed time-dependent nucleation rate across the WS_2_ basal plane using an extended form of Avrami’s classical nucleation equation to extrapolate an apparent overall nucleation barrier for oxidation of the given WS_2_ of approximately 1.1 eV. MLIP screening suggests that this barrier does not reflect the ease of initial oxygen incorporation, which is facile on many defect types with barriers often below 0.7 eV, but instead corresponds to the ability of a local environment to sustain sulfur removal, the step that commits the lattice to etching and governs pit propagation.^59^ Previous work has identified V_s_ and O_s_ as the most abundant defect types in such TMD layers, with densities of 10^12^ – 10^13^ cm^–2^ for typical CVD material, and V_w_ densities lower by more than 2 orders of magnitude.^35,37^ For the given reaction, however, the MLIP representation shows that for V_s_ and O_s_ sulfur-removal barriers exceed 1.5 eV, and therefore that these defects remain kinetically inert despite readily incorporating oxygen. In contrast, V_w_ complexes exhibit sulfur-removal barriers centered near the experimentally observed value and can act as effective nucleation sites for the reaction to propagate to the observed etching. Prior emphasis on initial O_2_ adsorption obscured the selectivity because this step is facile on most defects (see Figure S18).^29,61^ The decisive distinction emerges only at the point of sulfur removal, where abundant V_s_ and O_s_ defects prove inactive. The specific defect reactivity is thus critical here, not just mere abundance. This result also underscores the limits of typical static material characterization, which can only assess the abundance. Equally, this effect is critical when using propensity to etching as a proxy for defect density.

Our MLIP diffusion-barrier calculations indicate that both *V*_*S*_ and oxidized *V*_*W*_ configurations are practically immobile for our conditions (barriers >2 eV), precluding here scenarios in which such point defects migrate and aggregate to seed new pits.^66–69^ This conclusion implies that nucleation sites are consumed in place. The observed temporal evolution of the nucleation rate, characterized by an initial decrease followed by a plateau, can be rationalized by either (i) a progressive consumption of a population of pre-existing *V*_*W*_-like defects, partially balanced by the generation of new, active defect sites, or (ii) a superposition of nucleation dynamics arising from faster and slower reacting defect populations. These two scenarios may plausibly intersect, for example, sulfur–tungsten antisite defects (e.g., *S*_*W*_ or *S*_2*W*_) may exhibit slower nucleation kinetics but transform into *V*_*W*_-like configurations during progressive oxidation. We note, however, that the present analysis is limited to isolated point defects and focused on the basal plane in the middle of the WS_2_ domains, where we excluded edge regions, grain boundaries, multilayer regions or other larger defective areas that are discernible by SEM.

After nucleation, we find that pit growth can have time-dependent facet velocities. This dependence is incompatible with purely reaction-limited kinetics, which is an often made assumption and requisite for using kinetic Wulff plots and JMAK theory.^26^ For the pitting during oxidation of graphite, Langmuir–Hinshelwood mechanisms have been proposed,^70^ without, however, connecting to the atomic scale and discussing which surface species can be involved. Our phase-field modeling calibrated to *operando* observations shows indeed that this time dependence can arise from mobile surface species capable of generating concentration gradients across a length scale of the order of the size of the etch pits. MLIP diffusion barrier screening identifies chemisorbed hydroxyl species, with a diffusion barrier under 1 eV for hopping between neighboring surface sulfur atoms, as kinetically plausible under experimental conditions. OH-mediated pathways lead to formation of tungsten hydroxides, which are more volatile compared to tungsten oxides, a theme that dates back to historic W filament use.^71,72^ The mixed reaction regime is further characterized by high in-plane reaction anisotropy, arising from structure-dependent differences of the 1D reaction facets.^28,34^ The MLIP energy landscape reveals that sulfur-terminated zigzag edges exhibit O_2_ dissociation barriers consistently below 0.8 eV across all sulfur coverages, whereas tungsten-terminated zigzag edges show barriers exceeding 1.1 eV at moderate coverage and approaching 1.7 eV when fully sulfur-covered. This asymmetry directly quantifies the experimentally observed two- to 4-fold difference between fastest and slowest 1D facets at 450 and 680 °C, respectively. The origin is structural: tungsten-terminated edges favor sulfur-coordinated tungsten dimerization, which increases coordination and steric hindrance against oxygen activation, while sulfur-terminated edges lack this self-protective configuration. Desulfurization at tungsten-terminated edges therefore requires pre-existing defects or partial sulfur depletion, whereas sulfur-terminated edges activate readily. Beyond initiation, downstream removal and sublimation pathways are also 1D facet-specific. MLIP sampling of oxide-terminated edge configurations suggests that tungsten-terminated edges may undergo an unzipping-like process in which successive oxygen incorporation progressively breaks W–S bonds in outer atomic rows, effectively peeling back the edge, whereas sulfur-terminated edges follow distinct removal sequences with different intermediate configurations. These pathways are illustrative rather than unique, but they reinforce the fact that edge identity governs not only the onset of oxidation but also the subsequent removal chemistry and material loss.

As pits expand and merge, secondary facets and kinked boundaries emerge with distinct local coverages, diffusion access, and rate limiting steps. Pit morphology therefore encodes a complex kinetic history, rather than an equilibrium shape. Further, in this mixed reaction regime, the extrapolation of a single activation energy is not meaningful without contextualization into a specific reaction scenario.^21,40^ While our exploration here is by no means exhaustive in material, parameter, and coordination space, the fundamental insights gained enable an improved framework of understanding beyond the simple material model system. The rate balance between (defect-assisted) basal plane nucleation and anisotropic in-plane etching will, for instance, also be critical when looking at the etch behavior of multilayer WS_2_ or other 2D materials in general. An often invoked layer-by-layer etching is only feasible for a high in-plane etch rate and low basal plane nucleation rate, as otherwise the exposed basal plane of the second layer will start etching before the first layer has disappeared, and in the opposite scenario, etching of a multilayer will lead to triangular pyramid frustum shapes. The approach and insights presented are readily extendable to other chemical etchants and more complex coexposure scenarios, where spatiotemporal nucleation fingerprinting can be further refined as an assessment approach to sample quality. The understanding of the reactivity of different defect and edge types is highly relevant also for enabling effective defect reduction, passivation, modification, and doping strategies, as well as for assessing operational stability and lifetime of such device materials at monolayer level.

## Conclusions

We demonstrate a powerful hybrid approach to capture and explore multistep and multiscale reaction landscapes. We show that state-of-the-art MLIPs like MACE make atomistic exploration tractable while preserving first-principles fidelity. MLIPs enable the use of advanced sampling strategies, like basin-hopping global optimization in GMIN and discrete path sampling using OPTIM, to systematically explore complex multistep pathways and identify rate-limiting steps across full, previously intractable reaction sequences and the corresponding energy landscape. We show how this approach can be effectively combined with high-throughput experimental reaction screening by OSEM, which can capture the entire reaction progression from nanometer to millimeter scale. Our approach here is not to fall back to much slower, expensive, and complex experimental *operando* metrology with atomic resolution, but to collect required large, direct reaction data sets and drive a data-driven approach supported by classical continuum and phase-field modeling, which in turn can provide the required constraints to self-consistently guide the atomistic modeling. Taking the important model system of thermal oxidation of a monolayer semiconductor material, we show how this approach can readily reveal key reaction fingerprints and establish a deeper understanding of reaction kinetics. Because each tier constrains the next, our conclusions are synergistic, rather than additive. Our OSEM and modeling approaches are highly versatile and can readily be applied to reaction discovery and exploration across a wide horizon of materials and application areas. To support reuse, all data and code, including trained potentials, are made publicly available.

## Supplementary Material













## Notes

Description of experimental methods, further method details, additional sample characterization, XPS and OSEM data, derivations of Avrami nucleation and phase field model equations and MACE model training data and data sets (PDF)
Supplementary Video S1: OSEM of oxidation of monolayer WS_2_ at 680 °C (MP4)
Supplementary Video S2: OSEM of oxidation of monolayer WS_2_ at 450 °C (MP4)
Supplementary Video S3: Sulfur removal pathway near V_W_ modelled using the MACE potential and discrete path sampling (OPTIM) (MP4)
Supplementary Video S4: Sublimation pathway of WO_*x*_ species from the tungsten zigzag (W-ZZ) edge (MP4)
Supplementary Video S5: Sublimation pathway of WO_*x*_ species from the sulfur zigzag (S-ZZ) edge (MP4)
