# Supplementary material for: Accelerated Reaction Exploration across Scales: A Hybrid Operando and Modeling Study of Oxidation Kinetics in Monolayer Tungsten Disulfide
Source: J Am Chem Soc. 2026 May 20;148(21):21996–2007. doi: 10.1021/jacs.6c03511 (PMC13244466; doi:10.1021/jacs.6c03511)
Supplement: Supplementary file 1 [file ja6c03511_si_001.pdf]

# Supplementary Data

Accelerated Reaction Exploration across Scales: A Hybrid Operando and Modeling Study of Oxidation Kinetics in Monolayer Tungsten Disulfide

Jad Jaafar,[1,*] Ye Fan,[1,*] Maryam Kazemzadeh-Atoufi,[2] Ryo Mizuta,[1] Jinfeng Yang,[1] Jack E. N. Swallow,[3,4] Elizabeth Jones,[3] Matthijs Van Spronsen,[5] Georg Held,[5] Robert S. Weatherup,[3] Peter Voorhees,[2] David J. Wales,[6] Gabor Csanyi,[1] and Stephan Hofmann[1,+]

[1] Department of Engineering, University of Cambridge, Cambridge CB3 0FA, UK
[2] Department of Materials Science and Engineering, Northwestern University, Illinois, Evanston, IL 60208, USA [3] Department of Materials, University of Oxford, Oxford OX1 3PH, UK
[4] Department of Chemistry, University of Manchester, Manchester M13 9PL, UK
[5] Diamond Light Source, Didcot OX11 0DE, UK
[6] Department of Chemistry, University of Cambridge, Cambridge CB2 1EW, UK

*contributed equally

[+]corresponding author: sh315@cam.ac.uk

# Table of Contents

*I. Methods* ..... 2

i. **Sample Preparation** ..... 2

ii. **Operando SEM** ..... 2

iii. **Operando XPS** ..... 2

iv. **Further Characterisation** ..... 3

v. **Modelling** ..... 3

Phase Field Model ..... 3

Density Functional Theory Settings ..... 3

MACE Model Parameters ..... 4

Exploring the Energy Landscape: Basin-Hopping and Discrete Path Sampling ..... 4

*II. Setup and method details for in-situ oxidation in operando SEM (OSEM)* ..... 5

*III. SEM Image processing* ..... 9

*IV. Additional operando XPS data* ..... 10

*V. $WS_2$ characterisation before oxidation* ..... 11

*VI. $WS_2$ characterisation after oxidation* ..... 12

*VII. Additional kinetic data* ..... 16

*VIII. Derivation of extended Avrami nucleation equation* ..... 18

*IX. Derivation of Phase Field Model Equations* ..... 20

*X. Atomistic Simulations* ..... 23

i. **MACE Model Data Set and Training** ..... 23

ii. **Reaction Path Discovery and Barrier Calculations** ..... 25

iii. **Initial $O_2$ Chemisorption on various defects on $WS_2$** ..... 27

iv. **Initial Oxidation Near Various Sites on $WS_2$ for Sulfur Removal** ..... 29

v. **Sulfur Removal Pathway Computed using OPTIM** ..... 30

vi. Sulfur Removal Through SO Desorption ..... 31
vii. Diffusion Pathways on $WS_2$ Basal Plane ..... 32
viii. Initial $O_2$ reaction on W-ZZ and S-ZZ Edges Energy Profiles ..... 34
*XI. Supplementary Videos* ..... *37*
*XII. References* ..... *38*

# I. Methods

## i. Sample Preparation

The $WS_2$ domains were chemical vapour deposited (CVD) directly onto $SiO_2$ support to avoid transfer related contamination.[1] Monolayer $WS_2$ single crystals were grown on 300 nm $SiO_2$/Si substrate with sulfur powder (300 mg, >99.5%, Sigma-Aldrich) and $WO_3$ powder (200 mg, >99.5%, Sigma-Aldrich) as precursors. A two-furnace system was used to separately control the temperature of the two precursors. A smaller quartz tube was used to load the $WO_3$ powder to avoid sulfurization of the solid precursor and the substrate was placed 4 cm away from the open end of the small tube to allow sufficient mixing of the gaseous precursors. Sulfur was first heated to 180 °C to create a S-rich environment, while the $WO_3$ and substrate were heated (40 °C/min) to 1145 °C and 960 °C, respectively. The reaction was carried out for 4 min under 250 sccm Ar, after which the downstream furnace temperature was set to 0 and the gas flow was reduced to 10 sccm Ar. The sulfur was then heated to 400 °C in order to remove any remaining sulfur from the reaction zone. Finally, both furnaces were rapidly cooled to room temperature.[1]

## ii. Operando SEM

A high-resolution field emission ZEISS Gemini 300 SEM was used, and all images/videos shown were recorded with an In-Lens SE detector. Samples were loaded onto a Kammrath & Weiss 1050 Heating Module, with the temperature measured by a type-K thermocouple in direct contact with the top sample surface. Ambient air was introduced via a robotically actuated custom quartz micro-nozzle. SI Fig. S4 schematically summarises the experimental workflow using the micro-nozzle. Imaging was performed at a working distance of 12 mm, 5kV acceleration voltage and typical beam current of 675 pA. An entire image was scanned in ~3 seconds.

## iii. Operando XPS

Operando XPS measurements were conducted at the ambient-pressure Versatile Soft X-ray (VerSoX) beamline, B07 (branch C), at the Diamond Light Source.[2] To enable high-pressure operation, the sample chamber interfaces with the incoming synchrotron beam and the hemispherical electron analyser (SPECS, Phoibos 150 NAP) via differentially pumped sections. All $SiO_2$ supported $WS_2$ samples used for operando XPS measurements were prepared under the same conditions as for OSEM (see above). Samples were loaded in a small reaction chamber with a volume of 0.7 L (the 'T-cup'). Heating was provided by a button heater (HeatWave Labs) and local temperatures were measured by an internal thermocouple. All temperatures quoted here for operando XPS measurements correspond to corrected thermocouple readings, which have been calibrated based on melting tests of $Na_2MoO_4$ salt

(melting point 687°C). During oxidation, $O_2$ (~0.4 mbar) was dosed into the chamber via a piezo-controlled leak valve. All core level spectra were taken with a photon energy of 850 eV. The monochromator was operated with the 400 lines-per-mm grating, the exit slit was set to 50 μm in the dispersive direction, and the analyser pass energy was set to 40 eV, which leads to an overall energy resolution of approximately 0.5 eV. The footprint of the X-ray beam on the sample (at an incidence angle of 30° from the surface plane) was approximately 70 μm × 200 μm. Core level spectra were acquired with a pass energy of 40 eV and energy step size of 0.1 eV. The binding energy scale of W 4f and S 2p core level spectra was referenced to the O 1s core level from the $SiO_2$ substrate ($E_b$=533 eV) that was simultaneously measured. All fittings were performed using Voigt peak lineshapes and a Shirley background. For the fitting of core level doublets, the fitted peak areas were fixed to ratios of 4:3 and 2:1 for f and p core level spectra, respectively.

### iv. Further Characterisation

In order to minimise any unwanted photo-induced degradation of $WS_2$ under ambient conditions, all samples were stored under complete darkness.[3] All AFM measurements were performed using an MFP-3D AFM System (Asylum/Oxford Instruments) using tapping mode, at room temperature under ambient conditions. All AFM data was flattened and denoised using Gwyddion v2.55. Raman spectroscopy measurements were performed on a Renishaw InVia Raman microscope at 532nm excitation with a 100x objective lens using 1 or 2 mW laser power. Photoluminescence (PL) characterisation was performed on the same Raman microscope under the same settings except for a lower laser power of ~20 μW. Prior to analysis, the background was subtracted from all spectra using spline fitting in the Renishaw WiRE software. Raman and PL peaks were fitted using Lorentzian line-shapes. Any fresh $WS_2$ domains that were optically characterised were not used for thermal oxidation experiments.

### v. Modelling

Phase Field Model

A standard finite difference implementation of the phase field equations was used. Numerically stable calculations were performed by having a sufficiently small timestep. The interfacial width was chosen to be smaller than the smallest radius of curvature at the rounded corners of the triangular shapes. Parabolic energy curves as a function of O concentration of the bulk phases were employed. The computational boxes were chosen to be sufficiently large to ensure that boundary conditions on the edges of the boxes do not affect the results. The value of $\delta_L$ used was 0.99 such that there is strong reaction rate anisotropy that dominates over edge energy ansiotropy, replicating the sharp corners seen experimentally on the corrosion pits. $L_o$ was taken to be the kinetic coefficient that results in bulk-diffusion-controlled growth with an infinite reaction rate at the edge.[4]

Density Functional Theory Settings

DFT calculations were performed at the PBE-D3(BJ) level of theory as implemented in the CASTEP code.[5–9] The PBE functional has been shown to adequately describe structural markers of defected monolayer $WS_2$.[10] Calculations were performed using with periodic boundary conditions, a Monkhorst-Pack k-point grid,[11] plane-wave basis sets (cutoff: 700 eV),[12] and pseudopotentials for core electron treatment.[5] Pulay mixing was used to accelerate SCF convergence.[13] Convergence tests determined a k-point spacing of 0.1/Å falling within an energy/force difference criterion of 1 meV and 1 meV/Å, respectively. Geometry optimization

was conducted with a maximum force tolerance of 0.01 eV/Å, yielding a lattice parameter of 3.187 Å. All calculations were performed with spin polarization, with an initial spin of 1 assigned to the cell and the spin left unconstrained. This initial starting value was found to lead to the lowest energy configurations when a variety of edge models with different terminations were geometry-optimized starting from different initial spin values. ASE and wfl Python libraries were heavily used in preparing and submitting DFT calculations.[14,15]

MACE Model Parameters

The models trained in this work utilize hyperparameter settings that are synonymous to those of the MACE-MP-0 large model.[16] This configuration strikes a balance between expressiveness, accuracy, and computational efficiency, making it well-suited for fine-tuning models and for comparison with models trained from scratch, among other tasks. Specifically, the hyperparameters include two MACE layers, a spherical expansion up to $\iota_{max} = 3$, and 4-body messages in each layer (correlation order 3).[17] All models employ a 128-channel dimension for tensor decomposition. We use a radial cutoff of 6 Å and expand the interatomic distances into 10 Bessel functions, which are then multiplied by a smooth polynomial cutoff function to construct radial features. These features are subsequently fed into a fully connected feed-forward neural network with three hidden layers, each consisting of 64 hidden units, with SiLU as the non-linear activation function. The message equivariance is set to L= 2, in alignment with the MACE-MP-0 large model. The irreducible representations of the messages alternate in parity (using the e3nn notation, 128 ×0e+ 128 ×1o).

Exploring the Energy Landscape: Basin-Hopping and Discrete Path Sampling

The energy landscape was explored using tools based on geometry optimisation, which have been widely applied for molecules, condensed matter, and general optimisation problems. To harvest low-energy configurations we employed basin-hopping global optimisation as implemented in the GMIN code.[18–20] The basic algorithm involves accepting or rejecting moves in a chain of local minima, where the current minimum is perturbed and then minimised. A variety of approaches for the geometrical perturbations and acceptance criteria are available in GMIN, including a cyclic taboo list to provide a memory of previous structures. The organisation of the energy landscape was characterised by locating pathways between the minima of interest mediated by true transition states (stationary points with a single negative Hessian eigenvalue). This approach corresponds to discrete path sampling,[21,22] with transition states and multistep pathways calculated using the OPTIM program.[21–23] Transition state candidates are suggested using a doubly-nudged elastic band chain of images,[24–26] and accurately refined using hybrid eigenvector-following.[27–29] Approximate steepest-descent paths are calculated for each transition state to identify the corresponding minima. Any gaps in the pathway are then systematically filled in using the missing connection algorithm to select pairs of minima for the next round of transition state calculations until a complete path is obtained.[30] The end minima can be optimally aligned with respect to permutations of identical atoms using standard procedures in the GMIN, OPTIM, and PATHSAMPLE programs.[31,32] To treat dissociative processes we employed external potentials to pull selected atoms apart,[33] and to catch dissociating fragments.[34] These fields can be applied in both the GMIN and OPTIM programs. The external potentials were removed once suitable candidates for connection attempts had been identified, and do not affect the reported energetics.

## II. Setup and method details for in-situ oxidation in operando SEM (OSEM)

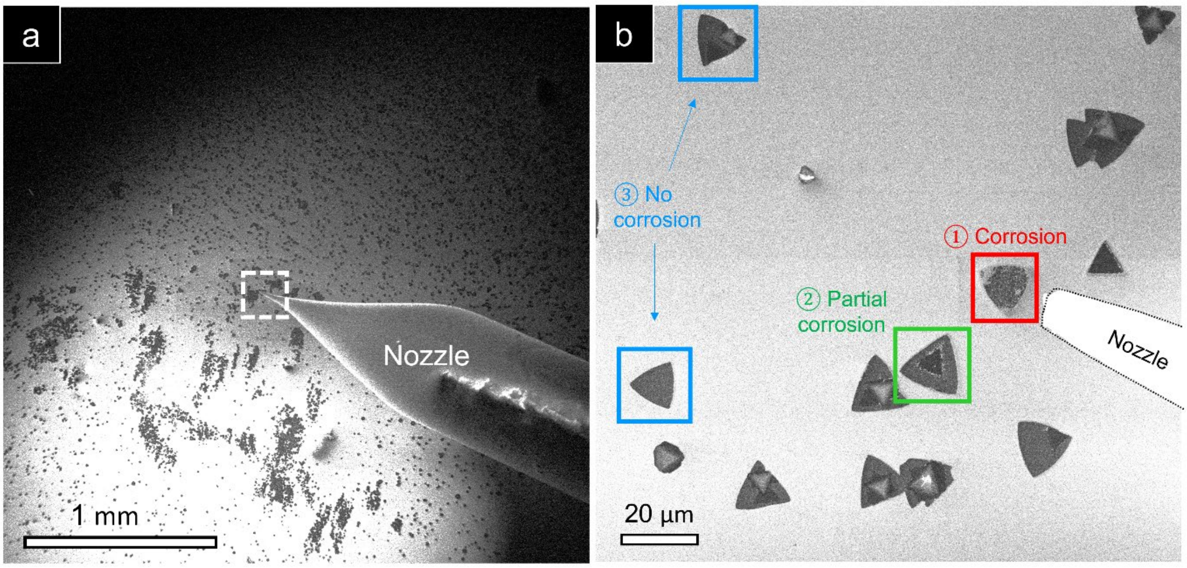


**Fig. S1 Localised gas injection setup. (a)** SEM image of the actuated quartz micro-nozzle. **(b)** SEM image showing the spatial localisation of $WS_2$ oxidation via gas injection through the micro-nozzle. The nozzle height was 50 μm and the exit diameter of the nozzle was 10 μm. The temperature was 680ºC.

**Fig. S1**(a) shows a low-magnification SEM image of the gas injection micro-nozzle used for the localised oxidation experiments. The exit diameter of the nozzle is comparable to the length-scale of a typical $WS_2$ domain, allowing for the flux of air to be highly localised. **Fig. S1**(b) illustrates the degree of oxidation across $WS_2$ domains at different distances from the gas injection nozzle. Here, oxidation was performed at 680ºC with a nozzle height of 50 μm from the substrate surface. The global partial pressure of air was $\sim 3\times10^{-5}$ mbar, similar to all other experiments reported in this study. Region 1 (red box) indicates the $WS_2$ domain that was selectively oxidised for this experiment. The domain lies between 10 to 20 μm from the nozzle exit and exhibits significant oxidation, both inside and at the edges of the monolayer domain. Region 2 (green box) highlights a domain at approximately 30 to 40 μm from the nozzle exit. The domain exhibits much less oxidation than in region 1. Notably, oxidation occurs preferentially at the boundary between the darker multi-layer at the domain centre and the surrounding monolayer. In contrast the monolayer itself remains more intact than in region 1, indicating that the local air pressure is significantly reduced. The preferential oxidation is to be expected, as the boundary between different layer numbers may possess greater defect concentration and corrode more readily. Finally, region 3 (blue boxes) highlight 2 domains that lie 100 μm or further from the nozzle exit. Neither domains exhibit any visible oxidation. In particular, the upper domain possesses multilayer regions much like region 2, but even the preferential oxidation at the layer boundaries is absent, indicating that the pressure is so low at this distance that oxidation does not occur within the given experimental timescales.

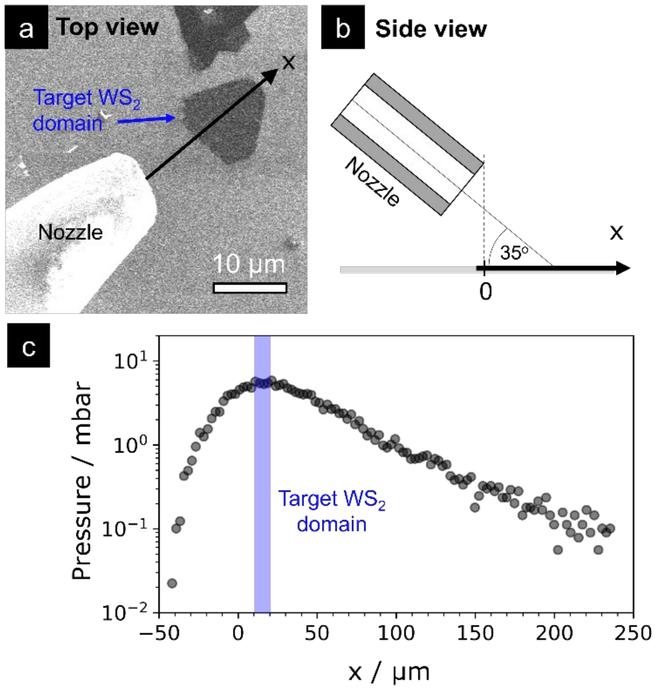


**Fig. S2 Simulation of pressure distribution of local gas injection via micro-nozzle. (a)** SE micrograph of the micro-nozzle configuration as viewed from above, showing its relative positioning to a single $WS_2$ domain. **(b)** Schematic showing the micro-nozzle configuration as viewed from the side. **(c)** Plot of expected pressure distribution at the substrate based on TPMC simulation. For the calculation, the nozzle height was 50 µm and the exit diameter of the nozzle was 10 µm. The gas particles were assumed to be $O_2$.

**Fig. S2**(a) shows an SEM image of a typical experimental situation for the operando oxidation. The exit diameter of the gas injection micro-nozzle is ~ 10 µm. The nozzle was positioned such that the target $WS_2$ domain was at approximately 10 µm from the edge of the nozzle (as viewed from above). The nozzle tip was held at a height of 50 µm from the substrate surface and an angle of ~35° to the horizontal, shown schematically in **Fig. S2**(b). For each experiment, the flow of air was adjusted and allowed to stabilise such that the global partial pressure of air inside the SEM was ~$3\times10^{-5}$ mbar. The corresponding local gas flux distributions were simulated using a MATLAB implementation of Test-Particle Monte-Carlo (TPMC) simulations, developed by Friedli et al.[35] In addition to the geometrical parameters of the nozzle positioning as described above, the simulation requires the flux of gas particles that exit the micro-nozzle, $J_{GIS}$ (particles $m^{-2}$ $s^{-1}$), to be defined a-priori. This may be calculated following the analysis reported by Sanz-Hernandez et al.[36]

Since the pressure inside the SEM was stable during gas injection, steady-state is assumed. According to the conservation of mass, the throughput of air particles that are introduced via the micro-nozzle, $Q_{GIS}$ (particles $s^{-1}$), equals the number of additional particles that are removed by the pump, $Q_{pump}$ (particles $s^{-1}$),

$$Q_{GIS} = Q_{pump}. \tag{1}$$

$Q_{pump}$ may be related to the measured partial pressure of injected air $P_{air}$ (Pa) according to

$$Q_{pump} = \frac{N k_B T}{P_{air}} \cdot S_{pump} \tag{2}$$

where N is the number of gas particles, $k_B$ is the Boltzmann constant, T (K) is temperature and $S_{pump}$ ($m^3$ $s^{-1}$) is the known volumetric extraction rate of the SEM pump. $J_{GIS}$ is then given by

$$J_{GIS} = \frac{4Q_{GIS}}{\pi d^2} \tag{3}$$

where d is the inner diameter of the nozzle at the exit. **Fig. S2** (c) illustrates the estimated pressure distribution at the substrate. The blue band highlights the approximate region of the $WS_2$ domain relative to the nozzle, over which a uniform pressure of ~6 mbar is calculated. Here, the calculations assume that the incident gas comprises of purely $O_2$. In contrast, ambient air was employed for the present experiments. Assuming that $O_2$ is the active oxidant in ambient air, the partial pressure of $O_2$ (21% of the simulated value) is ~1.3 mbar.

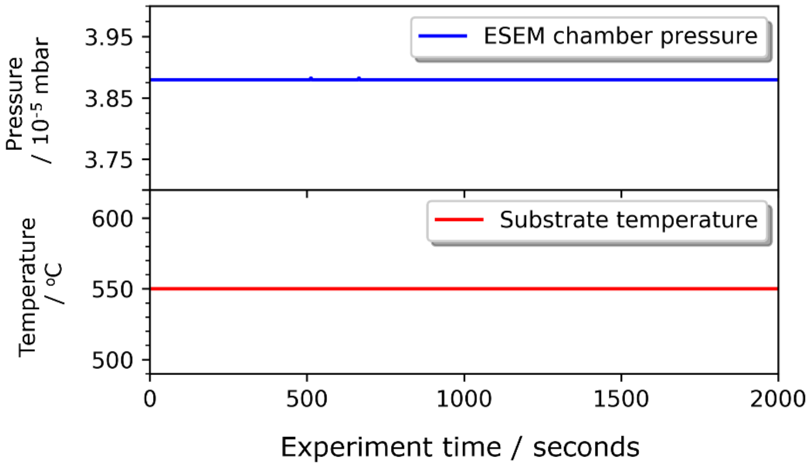


**Fig. S3 Stability of temperature and pressure during operando oxidation experiments.** Temporal stability of OSEM chamber pressure and substrate temperature. The representative data above was taken during operando oxidation at 550°C, with local gas injection, corresponding to ~$4\times10^{-5}$ mbar global pressure of air exposure. The plots demonstrate that oxidation conditions were highly stable during the operando experiments. The sample temperature is measured by a type K thermocouple placed on top of the sample substrate being oxidised. The OSEM chamber pressure was measured by a Penning gauge approx. 30 cm away from the localised gas injection site.

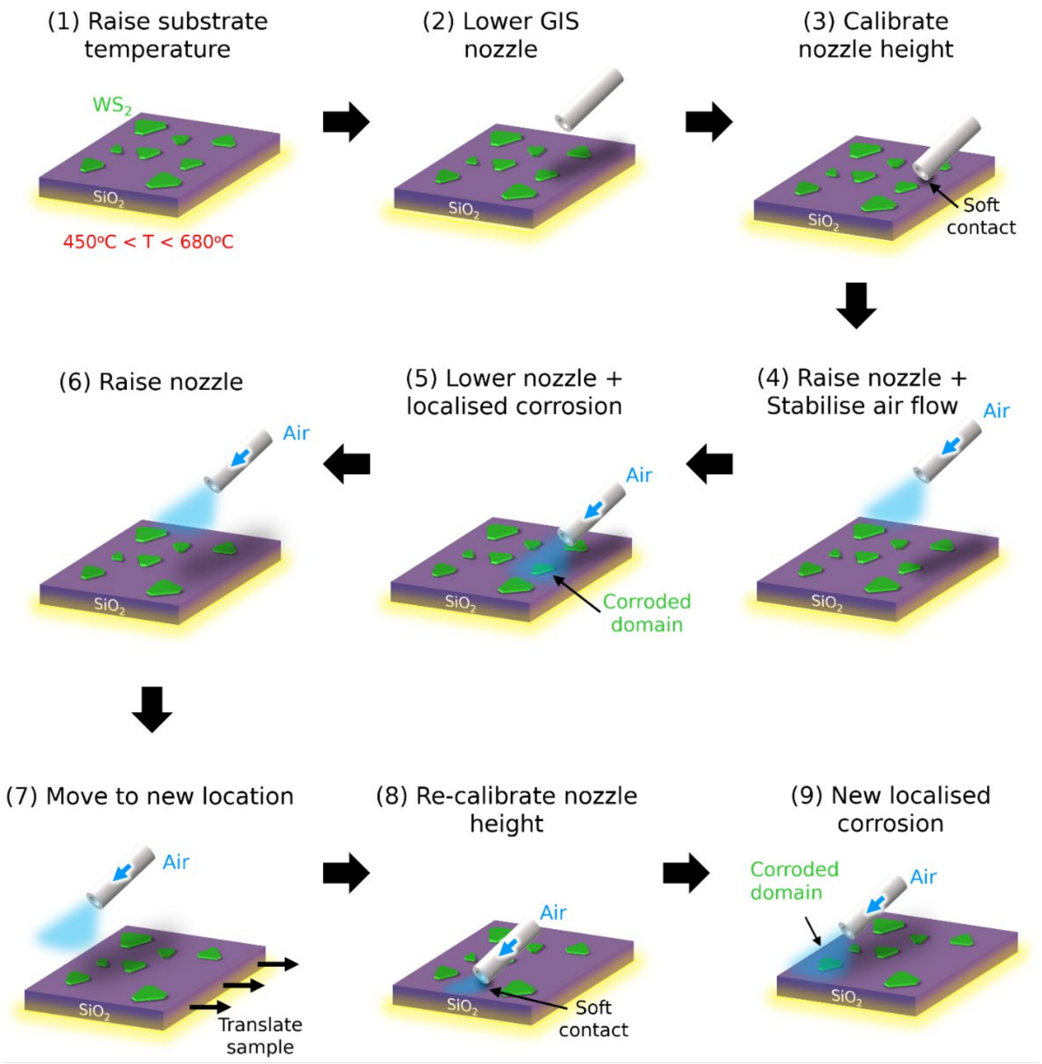


**Fig. S4 Experimental procedure for multiple localised oxidation experiments in high-throughput on the same sample substrate.**

The experimental steps for performing multiple operando localised oxidation experiments in the SEM using the same sample substrate are schematically shown in **Fig. S4**. **1:** The sample is heated to the target oxidation temperature using the heating module in vacuum (base pressure of SEM). **Step 2:** Once the target temperature was achieved, the gas injection nozzle was lowered to the sample. Note that this point, no air was being introduced yet. **Step 3:** The height of the nozzle tip above the sample substrate was calibrated and set. To calibrate, the nozzle was lowered until soft contact was achieved with the substrate surface. The nozzle was then raised to achieve the desired height, usually 50 µm. Note that this process could be performed very quickly, usually less than 1 min. **Step 4:** Air flow through the nozzle was introduced via a leak vale. During this step, the nozzle was raised to ~4 cm from the substrate surface (the maximum allowed by the SEM stage) as a precautionary measure. This significantly reduces the local air pressure at the substrate surface, allowing for any unwanted pre-oxidation of domains to be avoided before the air flow was stabilised. **Step 5:** Once stable air flow was achieved, the nozzle was lowered again to the working height established in step 3 and localised oxidation was started. Imaging was started immediately after the nozzle height was set. **Step 6 and 7:** After completing an oxidation experiment, the nozzle was raised and moved to a new location on the substrate for the next oxidation experiment. The raising of the nozzle was to limit any

unwanted oxidation as the nozzle was moved. The location of the nozzle was changed by laterally translating the sample relative to a stationary nozzle using the SEM sample stage control. Care was taken to select a fresh domain that was sufficiently far from any previous oxidation locations. **Step 8:** The nozzle is lowered and the same procedure as in step 3 is performed to rapidly re-calibrate and set the nozzle height. This step was included as a pre-cautionary measure to compensate for any unintentional changes to the nozzle height from the substrate. This may be due thermal drifting of the nozzle over time or the sample itself having non-uniform height. Note that this step is performed near but not at the location of the new domain, as air flow is still maintained and pre-oxidation of the domain of interest must be minimised. **Step 9:** The nozzle is moved to the new domain and the next oxidation experiment is started.

## III. SEM Image processing

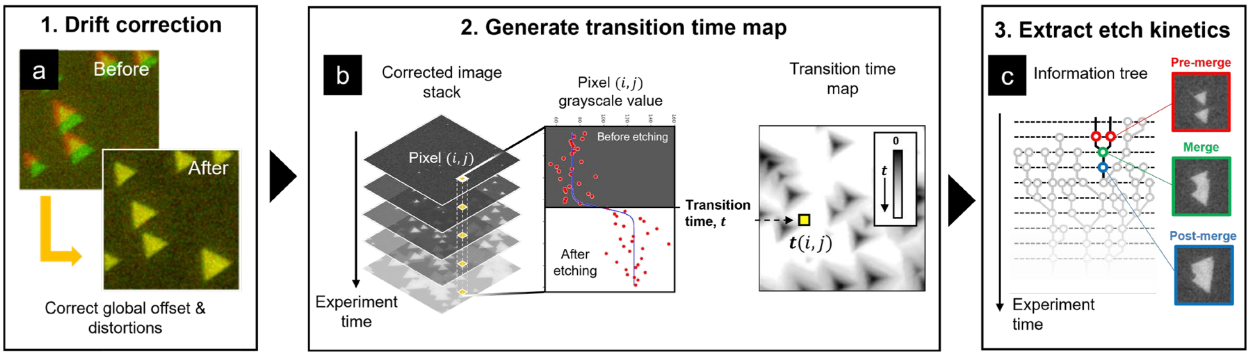


**Fig. S5 Workflow for OSEM image correction and data extraction.** (a) Drift correction, illustrated by a composite RGB image overlaying two typical consecutive images. The first image is coloured red and the second image is coloured green. The opacity is proportional to the grayscale of the original images, with a maximum opacity of 0.8. The SEM images have significant drift before the alignment (shown in upper image), while being aligned after the drift correction (shown in the lower image). (b) Transition time map. The oxidation of $WS_2$ is treated as a phase transition and the transition time for each pixel is mapped out. (c) extraction information and storage. The combination of etching pits is recognized and stored as an information tree.

The offset and distortion of SEM images are corrected by computer vison methods before extracting the materials growth information as shown in **Fig. S5**(a). Global image drift and frame by frame distortions are corrected for separately. The drift between frames is first aligned using phase correlation methods, which is referred as ‘global alignment’ hereafter. Subsequently, the distortions between each image are corrected by optimization-based image registration with a moving window, referred to as ‘local alignment’ hereafter. A representative image sequence before and after combined global and local alignments is shown in **Fig. S5**(a). Once the images are aligned, a characteristic etch “transition time”, t, is computed at each pixel location on the $WS_2$ domain. t corresponds to the experimental time that a given pixel’s value transitions from a dark value (secondary electron signal of monolayer $WS_2$, i.e. before etching) to light (signal of underlying substrate, i.e. after etching). It is extracted at each pixel location by fitting the pixel’s change in grayscale value over time with a hyperbolic tangent function. The resultant map of transition times, illustrated in **Fig. S5**(b), effectively captures when each section of $WS_2$ (contained within each pixel) underwent etching.

Our concept of transition times here is equivalent to the 'time of arrival of the crystal surface' that is originally discussed by Frank.[37] Despite having been originally proposed for theoretical discussion on geometric problems, our work demonstrates that a map of transition times neatly contains all the information on crystal nucleation and growth kinetics of interest. Following Frank's model, we extract "slowness" vectors, $\vec{s}$, from the transition time map according to

$$\vec{s} = \nabla t$$

Here, $\vec{s}$ is the direction-dependent rate at which an edge of an etch pit moves. The vector is parallel to the normal direction of the etch pit edge and also parallel to the reaction rate. The value of reaction slowness is the reciprocal of the reaction rate, $\vec{v}$, i.e. $|\vec{s}| \cdot |\vec{v}| = 1$.

The transition time maps are also used to perform intelligent image segmentation. The etched region at any given time is just the region whose phase transition time is less than t. This allows us to not only segment images into regions of etch pits and intact $WS_2$ but also helps to recognize the coalescence events between etching pits - see **Fig. S5**(c).

## IV. Additional operando XPS data

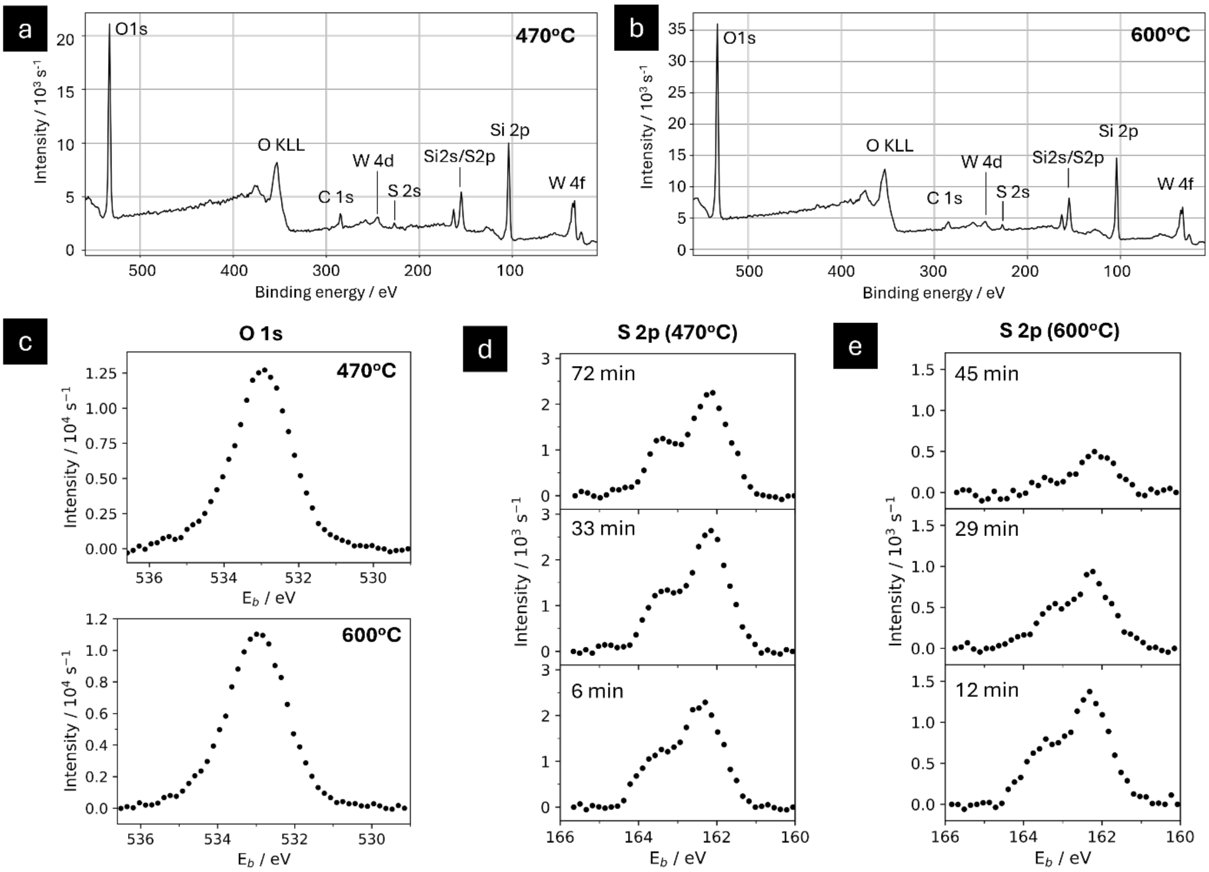


**Fig. S6** ***Operando XPS data of monolayer $WS_2$ oxidation*** *at ~0.4 mbar $O_2$ at 470°C and 600°C, measured alongside the W core level data shown in Figure 2. (a,b) XPS survey scans of the samples prior to their oxidation. Note: Si 2s and S 2p peaks at ~150 eV and ~160 eV, respectively, are seen to overlap in our survey spectra due to the poorer energy resolution at the higher pass energy used, but do not overlap in high-resolution scans measured with lower pass energies. (c) Representative O 1s core level spectra. The O 1s peak, originating predominantly from the $SiO_2$ substrate, was used in both cases as the binding energy reference*

*for each measurement cycle across all core level spectra. Its position was set to 533 eV, consistent with reported O 1s binding energies for $SiO_2$. (d,e) Process time-dependence of S $2p_{1/2}$ and $2p_{3/2}$ doublet peaks at 470°C and 600°C, respectively. The S $2p_{1/2}$ and $2p_{3/2}$ peaks were observed at BEs of 163.4 and 162.1 eV, respectively, at 470°C, and 163.5 and 162.2 eV, respectively, at 600°C, in agreement with reported S 2p peak positions in $WS_2$.[38] For all spectra background subtraction has been applied via fitting of a Shirley function.*

## V. $WS_2$ characterisation before oxidation

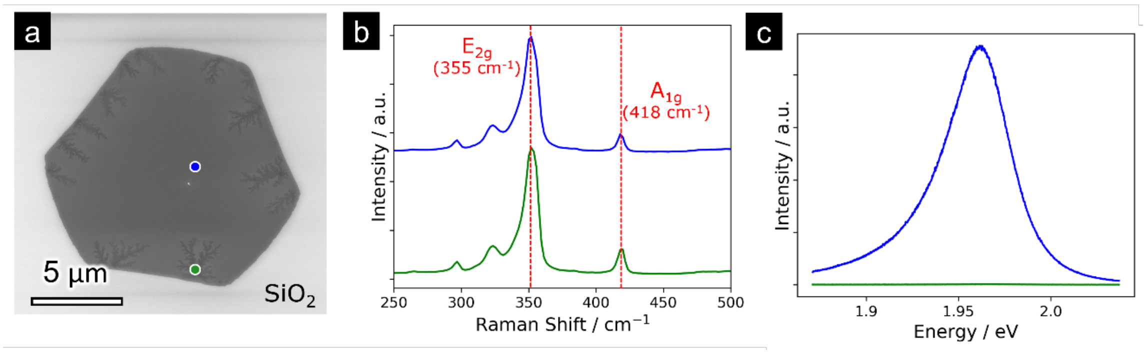


**Fig. S7 Raman and Photoluminescence (PL) characterisation of $WS_2$ domains prior to oxidation. (a)** SEM image of a typical $WS_2$ domain. **(b)** Raman and **(c)** PL spectra taken from the centre (blue curve) and dendrite at the edge of the $WS_2$ domain (green curves). Corresponding locations are indicated by blue and green dots in (a), respectively. The strong PL signature at the centre of the domain indicates a monolayer. Meanwhile, the quenched PL signal in the spectrum taken from the dendrite at the domain edge indicates a multilayer, as expected. Both Raman and PL spectra were collected with 532 nm excitation laser at 10 µW.

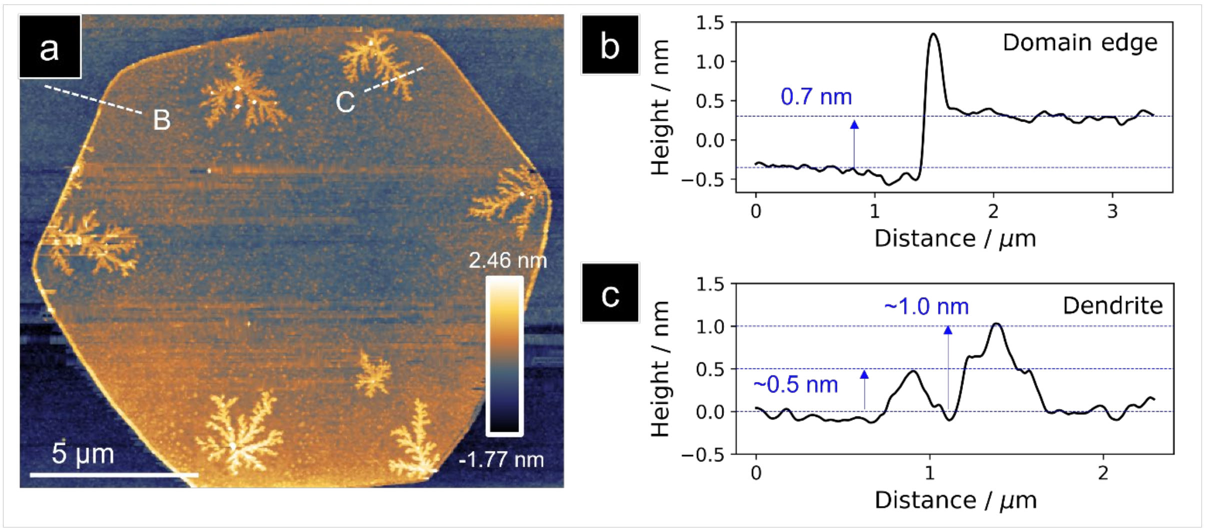


**Fig. S8 AFM characterisation of monolayer $WS_2$ domain before thermal oxidation (a)** AFM image of a full representative $WS_2$ domain. Height profiles are of **(b)** the domain edge and **(c)** over a dendrite region, indicated by dashed white lines and corresponding labels in (a).

## VI. $WS_2$ characterisation after oxidation

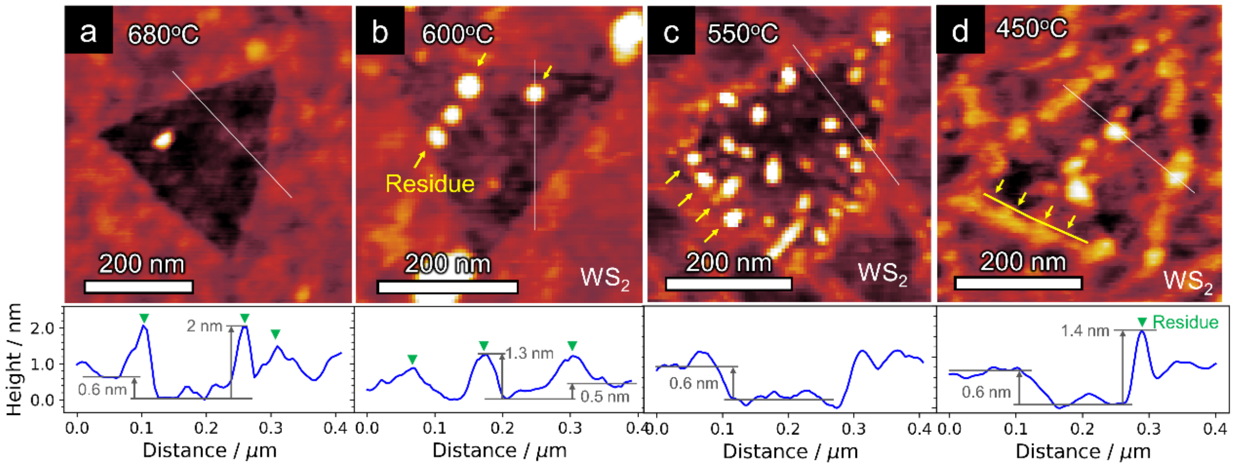


**Fig. S9 AFM images of $WS_2$ after thermal oxidation** at four different temperatures using localised air injection in the OSEM. The white lines indicate position of respective height profiles. Yellow arrows and text highlight residues of W oxides post-oxidation within the etch pit.

Post-oxidation atomic force microscopy (AFM) and the corresponding height profile in **Fig. S9**(a) show that the SEM contrast features correspond to holes in the $WS_2$ domains, with the measured depth of 0.6 nm being consistent with the monolayer $WS_2$ thickness. The feature morphology significantly changes for lower oxidation temperatures with all conditions otherwise unchanged (**Fig. S9**b-d), specifically the emergence of globular residues is seen, 1-2 nm in height and up to 10s of nm in lateral dimensions. For 550°C a relative increase in such globular residues is observed, within the increasingly less well-defined hole edges. This trend is further amplified at 450°C (**Fig. S9**d), where particularly also the hole edges are decorated with residue. Despite the increased amount of residue, the lateral extent of the depressions formed remains clearly distinguishable at 450°C both in the AFM and SEM signatures. AFM analysis confirms that the observed oxidation features are etch pits. Comparing AFM and SEM images for different temperatures allows us to conclude that the residual W oxide decoration has minimal impact on the SE feature contrast. Consequently, SE analysis enables clear tracing of basal plane nucleation and in-plane expansion of 1D reaction fronts, which collectively constitute the overall reaction.

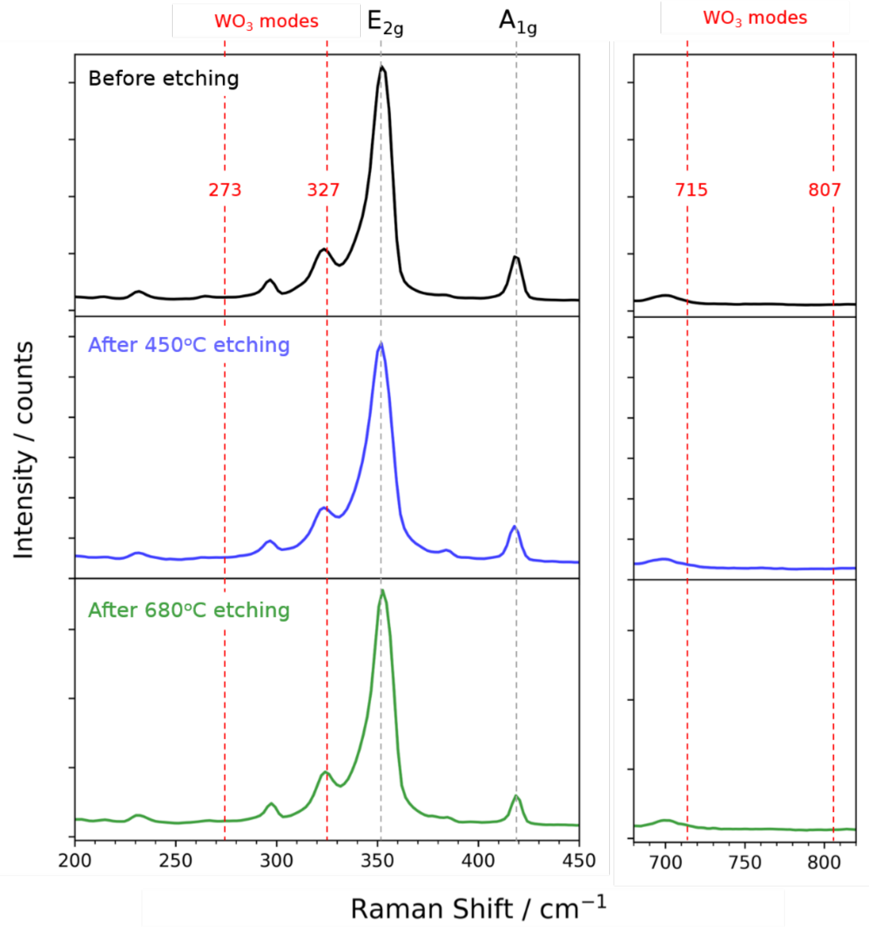


**Fig. S10 Raman spectra of $WS_2$ monolayer domains before and after thermal oxidation** at 450°C and 680°C using localised air injection in the OSEM.

**Fig. S10** compares the Raman spectra of $WS_2$ domains before and after etching at 450°C and 680°C. Note that the oxidation was partial, i.e. oxidation was stopped before the entire domain was etched such that there was still residual $WS_2$. The characteristic $E_{2g}$ and $A_{1g}$ peaks of $WS_2$ could be observed even after oxidation at ~352 and ~418 $cm^{-1}$, respectively, which are unchanged from those in $WS_2$ Raman spectra before oxidation. Furthermore, no significant peak broadening could be seen after oxidation, indicating that the crystallinity of the remaining $WS_2$ remained high.

The dashed red lines indicate the expected Raman peak positions for $WO_3$, the main anticipated product of the oxidation reaction for $WS_2$.[39] For the oxidation of extremely thick $WSe_2$ single crystals, clear $WO_3$ peaks were reported at 328, 707, and 804 $cm^{-1}$.[40] However, no clear indication of $WO_3$ could be seen even for oxidation at 450°C. Note however that this does not necessarily indicate the absence of $WO_3$, and can also be attributed to the $WO_3$ being highly amorphous and/or its quantity being below the detection limit.[41]

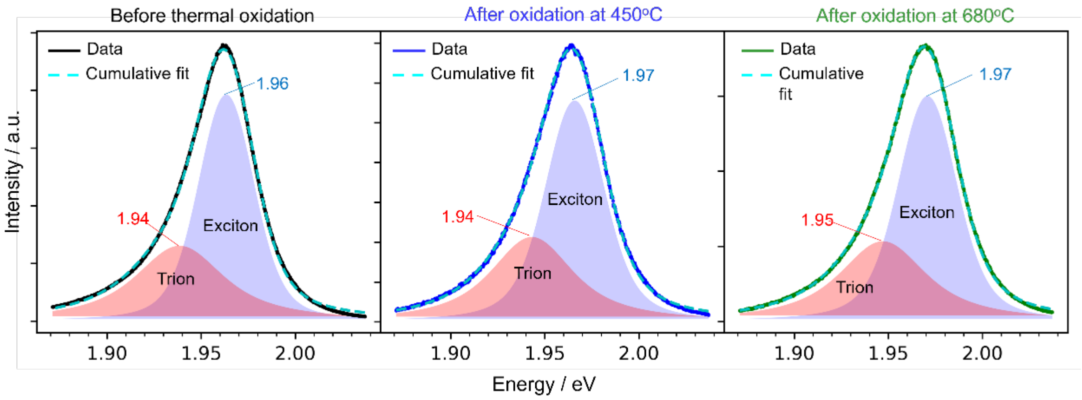


**Fig. S11 Representative PL spectra taken from $WS_2$ monolayer domains before and after OSEM** of thermal oxidation with locally injected air at 450°C and 680°C. Exciton and trion components are extracted by fitting with a two-component Voigt line-shape. Spectra were collected from the centre of domains in monolayer regions (as performed in **Fig. S6)**, avoiding domain edges and any small bilayer/multilayer dendrites around the domain periphery.

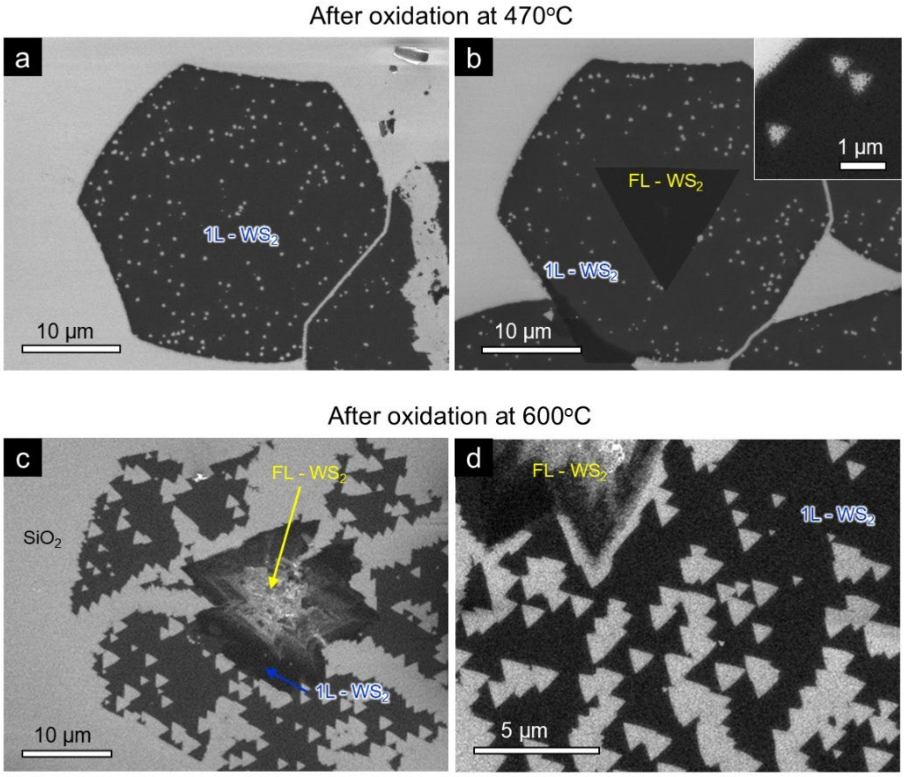


**Fig. S12 SEM of $WS_2$ domains on $SiO_2$ after operando XPS measurements** of thermal oxidation at ~0.4 mbar $O_2$ at (a,b) 470°C and (c,d) 600°C. 1L = monolayer. FL = few layer. Note that 1L $WS_2$ regions are noticeably oxidised while FL regions; preferential oxidation of the 1L is particularly evident in (b). Also note that anisotropic triangular oxidation features are observed, in agreement with results obtained by OSEM. In (b) inset, dark deposits are also noted lining the internal edge of the triangles, attributed to deposits of $WO_3$ residue.

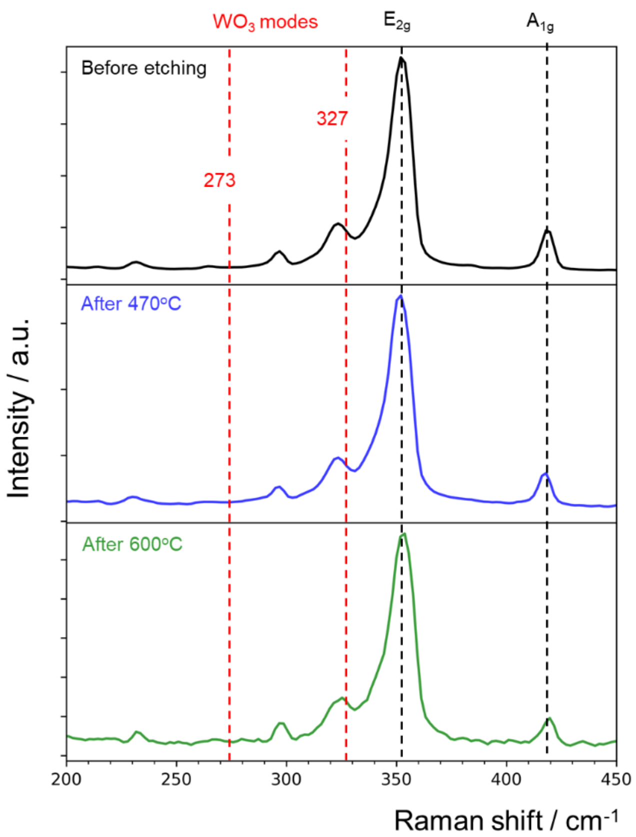


**Fig. S13 Raman spectra taken from intact regions of $WS_2$ monolayer between oxidation pits after operando XPS** at ~0.4 mbar $O_2$. A reference spectrum of pristine $WS_2$ before any oxidation is also included. No significant broadening, shifting or loss in peaks is observed, indicating that the $WS_2$ between oxidation holes is still crystalline $WS_2$. This is consistent with our post-oxidation analysis of samples studied by OSEM (Fig. S9).

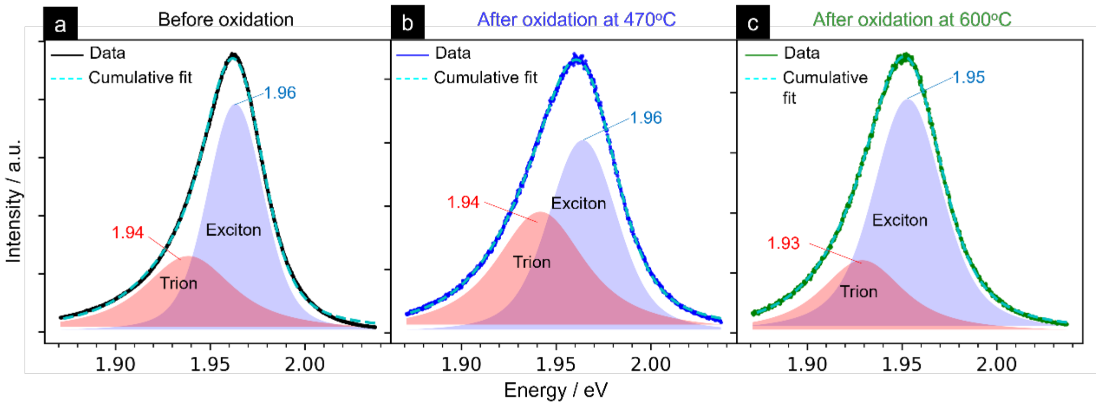


**Fig. S14 Representative PL spectra taken from intact regions of $WS_2$ monolayer domains after operando XPS measurements** of thermal oxidation at ~0.4 mbar $O_2$**.** A reference spectrum of pristine $WS_2$ before any oxidation is also included. Exciton and trion components are extracted by fitting with a two-component Voigt line-shape. Spectra were collected from the centre of domains in monolayer regions, avoiding domain edges and any small bilayer/multilayer dendrites around the domain periphery.

## VII. Additional kinetic data

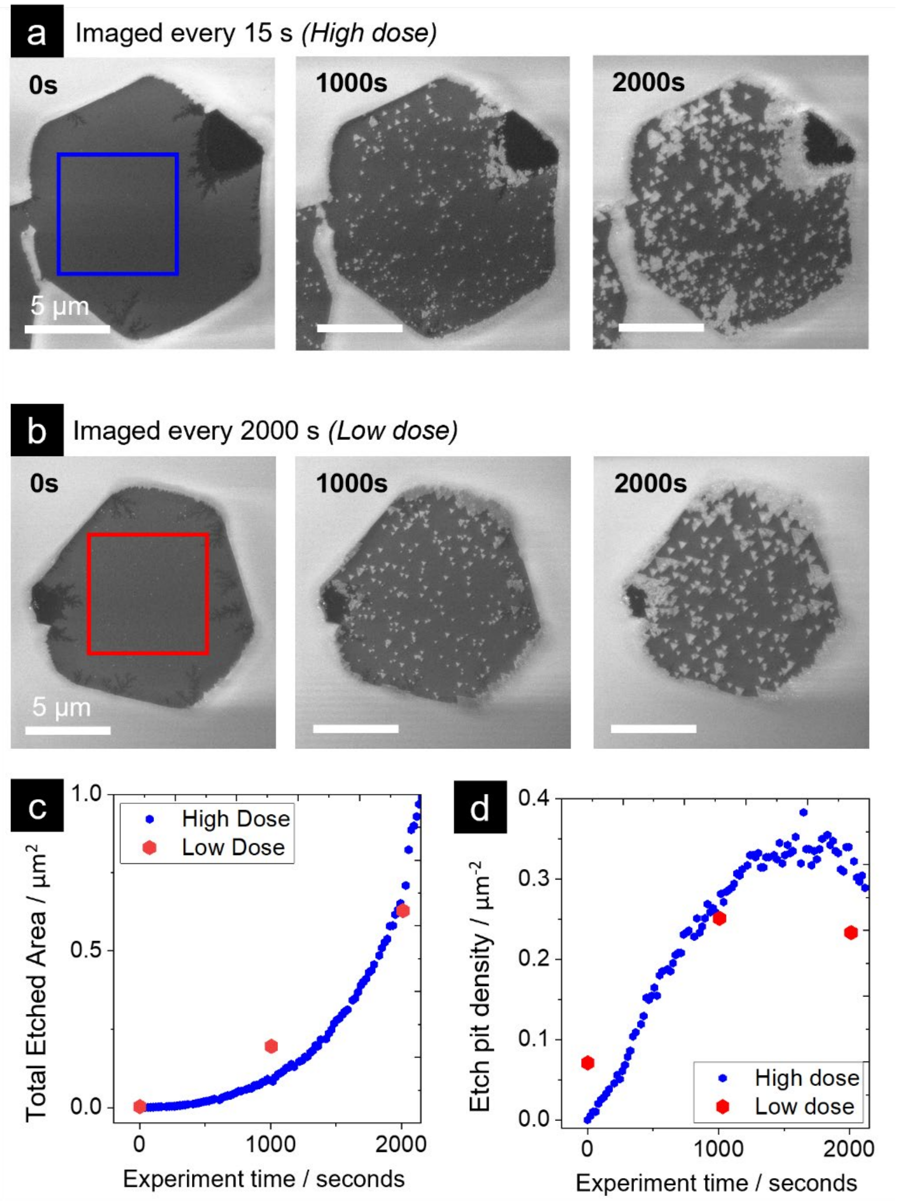


**Fig. S15 Impact of electron beam on oxidation kinetics. (a,b)** OSEM images taken after 0, 1000 and 2000 seconds after the start of oxidation using localised air injection, when imaging was performed **(a)** every 15 seconds (high-dose), and **(b)** every 1000s seconds (low-dose). **(c)** The total oxidised area and **(d)** density of etch pits taken from monolayer regions in (a) and (b), indicated by the blue (high dose) and red (low dose) boxes.

The influence of the electron beam on the oxidation kinetics was studied by conducting equivalent oxidation experiments with different cumulative beam doses. Both domains in **Fig. S15**(a) and (b) were oxidised at 550ºC in ~1.5 mbar (local pressure) of air. The beam current and acceleration voltage were 675 pA and 5kV (the same as for all data in this study). In **Fig. S15**(a), the domain was imaged every 15 seconds. In **Fig. S15**(b), the domain was only imaged every 1000 seconds. These two conditions are denoted high-dose and low-dose respectively. Each image covered an area of 20μm x 20 μm, and took 3.9 seconds to acquire with a beam dose of $4.2 \times 10^7$ $e^- / \mu m^2 s^{-1}$. After 2000 seconds of imaging, the total doses were $1.2 \times 10^8$ and $4.2 \times 10^9$ $e^- / \mu m^2$ for the low-dose and high-dose domains, respectively.

Comparing **Fig. S15**(a) and (b) illustrates that there was no marked difference in the oxidation between high and low doses. This was confirmed by comparing the time-dependence of total etched area and etch hole density for the two doses, shown in **Fig. S15**(c) and (d) respectively. Note that these plots were extracted from exclusively monolayer regions, indicated by the blue (high-dose) and red (low-dose) boxes in **Fig. S15**(a) and (b) respectively. Given that sparsity of data-points from the low-dose condition, only general trends are highlighted. Nonetheless, **Fig. S15**(c) shows that under both dose conditions, the rate at which $WS_2$ is etched away are very similar, indicating that the beam plays a minimal role in the mechanism by which $WS_2$ is removed. **Fig. S15**(d) also shows that both dose conditions yield 1) comparable etch hole densities, and 2) similar rate of formation of etch holes. Therefore, it is also concluded that the electron beam does not significantly promote the nucleation of holes. Slight discrepancies in the absolute hole densities are attributed to variations in the starting defect density between domains.

## VIII. Derivation of extended Avrami nucleation equation

In Avrami's theory of nucleation, the original phase (here, intact monolayer $WS_2$) before any transformation has begun (i.e. oxidative etching) is assumed to have a set number of possible nucleation sites. During nucleation, the number of possible nucleation sites decrease over time by two possible mechanisms: the site activates and becomes a growing nucleus, or the site is eaten up by the growing new phase. For 2D nucleation,

$$dN = -dN' - dN'' \tag{1}$$

where N is the number of possible nucleation sites per unit area at time t, N′ is the number of active growth nuclei per unit area at time t, and N″ is the number of possible nucleation sites per unit area that are consumed by the growing new phase up to time t. In all these definitions, the variable is normalized by the initial area of the phase. Thus Equation 1 states that the number of possible nucleation sites decreases by the number of sites that nucleate and by the number of sites eaten up by the growing phase, up to time t.

The number of sites that become active growth nuclei is given by

$$dN' = nNdt \tag{2}$$

where n is the probability of a possible site forming into a growth nucleus per possible nucleation site per unit time.

The number of sites consumed by the growing new phase is given by

$$dN'' = \frac{N}{1-A}dA \tag{3}$$

where $1 - A$ is the fraction of untransformed area, and A is the fraction of transformed area (Here, both quantities are per unit area, normalized by the initial area before nucleation begins). dA is the change in area of the new phase per unit area of space in time dt.

Solving this system of equations leads to Avrami's result, below.

$$\frac{dN'}{dt} = \bar{N}[1 - A(\tau)]e^{-nt} \tag{4}$$

$$J = \frac{1}{1 - A(\tau)}\frac{dN'}{d\tau} = \bar{N}e^{-nt} \tag{5}$$

where J is the nucleation rate and $\bar{N}$ is the number of possible nucleation sites per unit area at $t = 0$, i.e. $\bar{N} \equiv N(0)$.

Here we expand on Avrami's theory by positing that possible nucleation sites are added during nucleation.

$$dN = -dN' - dN'' + dN''' \tag{6}$$

where N‴ is the number of possible nucleation sites added and is given by

$$dN''' = k(1 - A)dt \tag{7}$$

where k is positive and represents the rate of addition of possible nucleation sites during the experiment. Then equation (6) becomes

$$dN = -nNdt - \frac{N}{1-A}dA + k(1-A)dt \tag{8}$$

Rearranging yields a differential equation.

$$\frac{1}{N}dN + ndt + \frac{1}{1-A}dA - \frac{k}{N}(1-A)dt = 0 \tag{9}$$

$$\frac{1}{N}\frac{dN}{dt} + \frac{1}{1-A}\frac{dA}{dt} + n - \frac{k}{N}(1-A) = 0 \tag{10}$$

Defining a density $\rho(t)$

$$\rho(t) = \frac{N(t)}{1-A(t)} \tag{11}$$

$$\frac{1}{\rho(t)}\frac{d\rho(t)}{dt} + n - \frac{k}{\rho(t)} = 0 \tag{12}$$

Integrating, gives the number density of nuclei,

$$\rho(t) = \left(\bar{\rho} - \frac{k}{n}\right)e^{-nt} + \frac{k}{n} \tag{13}$$

where $\bar{\rho}$ is the initial density of possible nucleation sites. The nucleation rate J per area of untransformed region is

$$J = \frac{1}{1-A}\frac{dN'}{dt} \tag{14}$$

Note that this definition is slightly different than Avrami's since it contains $(1-A)$, a time-dependent area. From the definition of N′,

$$dN' = nNdt \rightarrow \frac{dN'}{dt} = nN \tag{15}$$

Using Equation 11 in Equation 15,

$$\frac{dN'}{dt} = n(1-A)\rho \tag{16}$$

Using Equation 16 in Equation 14 and Equation 13, The nucleation rate is:

$$J = n\left(\bar{\rho} - \frac{k}{n}\right)e^{-nt} + k \tag{17}$$

If $k/n \ll \bar{\rho}$, or the rate of addition of possible sites relative to the probability of the possible sites nucleating is small compared to the initial density of possible sites, then the nucleation rate decays to k. Defining an incubation time as $\tau = n^{-1}$, gives Equation 1 in the text.

## IX. Derivation of Phase Field Model Equations

To rationalize the complex mesoscale reaction kinetics observed, we use a quantitative phase-field model for a multicomponent, multi-phase system to simulate the etch pit growth, based on work by Moelans.[4] We propose that growth occurs by injection of an oxygen-containing species from the gas nozzle to the surface and by that species' diffusion along the surface of the $WS_2$ monolayer to the pit. We apply a binary multi-phase order parameter model with three phases ($WS_2$, $WO_x$, gas) in two dimensions.

For a multi-phase system with n components, the composition is represented by $n-1$ independent compositions $\vec{c}$. In our binary model, the diffusing species is oxygen-containing. The phases are represented by p non-conserved order parameters:

$$\vec{\eta} = \begin{pmatrix} \eta_1 \\ \vdots \\ \eta_p \end{pmatrix}$$

The order parameters (or phase variables) track the phases, such that within phase $\rho$, $\eta_\rho = 1$ and $\eta_\sigma = 0$ for all $\sigma \neq \rho$. The phase variables sum to one at each position. The diffuse interface is a narrow region where the order parameter continuously and steeply changes from 1 to 0.

The free energy of the system is given as

$$\mathcal{F} = \int_V \left\{ m f_0(\vec{\eta}) + \frac{\kappa}{2} \sum_\rho \left(\nabla \eta_\rho\right)^2 + \sum_\rho \phi_\rho f_\rho(c_\rho) \right\} dV$$

The first and second terms of the free energy functional comprise energy associated with the interface and are the multi-well potential and gradient energy contribution respectively. The multi-well potential sets the phase variable minima at 0 and 1 such that bulk phases minimize the energy of the system. The model parameter m can be thought of as the well height between the minima. The gradient energy coefficient $\kappa$ contributes positive energy with gradients in the phase variables. The balance between the multi-well potential and gradient energy contribution results in the diffuse interface as the equilibrium shape of the interface. The form for the multi-well potential proposed by Moelans is a 4th-order Landau polynomial:

$$f_0(\vec{\eta}) = \sum_\rho \left( \frac{\eta_\rho^4}{4} - \frac{\eta_\rho^2}{2} + \frac{3}{4} \sum_\rho \sum_{\sigma \neq \rho} \eta_\rho^2 \eta_\sigma^2 + \frac{1}{4} \right)$$

The last term of the energy functional $\mathcal{F}$ is the chemical bulk free energy density, an interpolation of the bulk pure free energy densities of each phase, $f_\rho$ for phase $\rho$. The interpolation functions are

$$\phi_\rho = \frac{\eta_\rho^2}{\sum_\rho \eta_\rho^2}$$

and can be thought of as phase fractions as they sum to 1. The chemical bulk free energy densities are evaluated at the virtual compositions $c_\rho$. These are calculated by the constraint that the diffusion chemical potentials at the virtual compositions are equal, i.e. the parallel tangent construction:

$$\tilde{\mu} = \frac{\partial f_\rho(c_\rho)}{\partial c_\rho} \quad \forall \rho$$

where $c_\rho$ is the virtual composition corresponding to phase $\rho$. The virtual compositions are related to the composition field by

$$c = \sum_\rho \phi_\rho c_\rho$$

These compositions are virtual since the bulk free energy densities are evaluated at these compositions across all points in the system, including the interface. The chemical bulk free energies are approximated as parabolic, which allows for the derivation of analytical expressions for the virtual compositions thereby increasing the computational efficiency of the model.

Minimization of the free energy functional $\mathcal{F}$ yields evolution equations for the composition field and order parameter fields. Each non-conserved order parameter is evolved by an Allen-Cahn equation:

$$\frac{\partial \eta_\rho}{\partial t} = -L(\vec{\eta}) \frac{\delta \mathcal{F}}{\delta \eta_\rho}$$

where $L(\vec{\eta})$ is the kinetic coefficient, related to interface mobility. The reaction rate at the $WS_2$ interface with gas or oxide depends on the inclination of the interface $\phi$, which is captured by an anisotropic interfacial mobility in the kinetic coefficient, $L(\phi)$.

$$L(\phi) = \frac{\sum_\rho \sum_{\sigma \neq \rho} L_{\rho,\sigma}(\phi) \eta_\rho^2 \eta_\sigma^2}{\sum_\rho \sum_{\sigma \neq \rho} \eta_\rho^2 \eta_\sigma^2}$$

where $L_{\rho,\sigma}(\phi)$ has the standard form for three-fold symmetry.

$$L_{\rho,\sigma}(\phi) = L_0\left(1 - \delta_L c \cos\left(3\phi_{\rho,\sigma}\right)\right)$$

For the $WS_2$ phase, the Allen-Cahn equation is

$$\frac{\partial \eta_{WS_2}}{\partial t} = -L(\vec{\eta}) \Bigg( m\left[\eta_{WS_2}^3 - \eta_{WS_2} + 3\eta_{WS_2}\left(\eta_{WO_x}^2 + \eta_{gas}^2\right)\right] - \kappa \nabla^2 \eta_{WS_2} + \frac{2\eta_{WS_2}}{\eta_{WS_2}^2 + \eta_{WO_x}^2 + \eta_{gas}^2}\left[\left(1 - \phi_{WS_2}\right)\left(f_{WS_2} - \tilde{\mu} c_{WS_2}\right) - \phi_{WO_x}\left(f_{WO_x} - \tilde{\mu} c_{WO_x}\right) - \phi_{gas}\left(f_{gas} - \tilde{\mu} c_{gas}\right)\right] \Bigg)$$

The concentration field evolution equation is based on an Onsager-type diffusion equation, where diffusion occurs along gradients in chemical potential [ref]. To simulate air exposure via

the gas injection nozzle, a source of oxygen (final term) was added to the diffusion equation for the O concentration,

$$\frac{\partial c}{\partial t} = \nabla \cdot \left[\{\phi_{WS_2} M_{WS_2} + \phi_{WO_x} M_{WO_x} + \phi_{gas} M_{gas}\} \nabla \tilde{\mu}\right] - Q\left(c - c_{\infty}\right) \eta_{WS_2}^{2}$$

where $c$ is the concentration in mole fraction of the oxygen-containing species and $M_\rho$ is the chemical mobility of the oxygen-containing species in the $\rho$ phase, following a mixture rule. The final term simulates the source of oxygen from the gas nozzle. The parameter $Q$ is the rate constant of the source term with units of number of species per second, and $c_{\infty}$ is the saturation level of oxygen-containing species on the surface of monolayer $WS_2$. Near the etch pit, $c < c_{\infty}$ and there is a flux into the $WS_2$ surface from the source term. Far from the etch pit $c = c_{\infty}$, and there is no flux.

## X. Atomistic Simulations

### i. MACE Model Data Set and Training

The interatomic potential for the $WS_2$–O–H system was developed using an iterative procedure designed to capture the complex energy landscape of $WS_2$ oxidation and sublimation. This approach ensured that the model remained robust across diverse chemical environments, from pristine phases to highly non-equilibrium transition states. The final dataset comprises 4,520 unique atomic configurations. This set was partitioned into a training/validation pool of 4,181 structures and an independent, external test set of 339 structures. Within the training pool, 3,972 configurations were utilized for parameter optimization, while 209 (5%) were reserved for internal validation and hyperparameter monitoring.

**Structural Diversity**

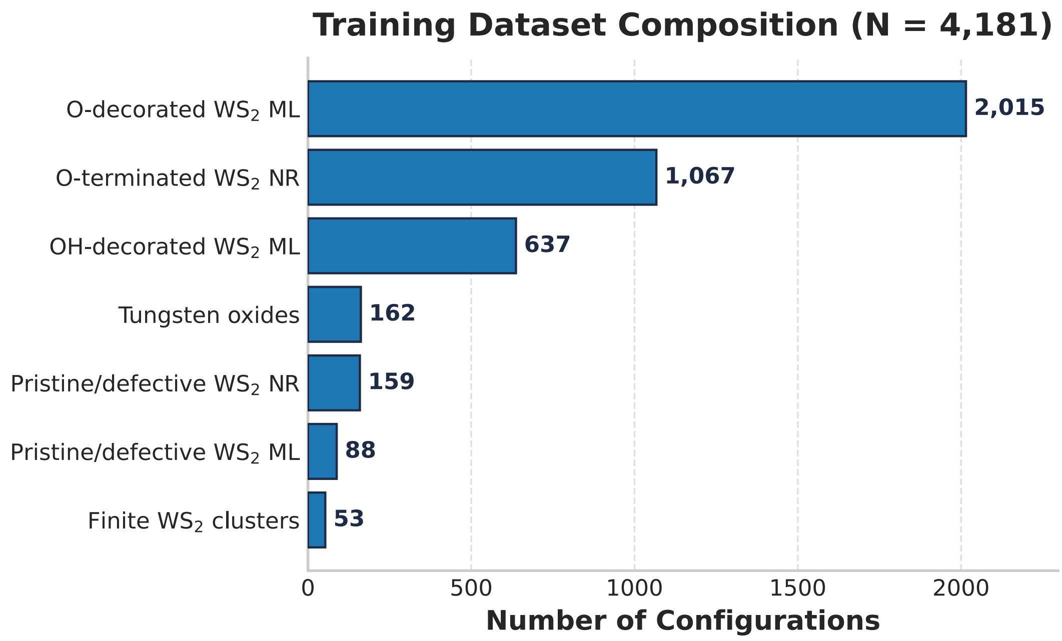


**Fig. S16 Training dataset distribution across key chemical environments.** Training dataset composition (N = 4,181) across WS2 structure classes: O-decorated monolayers (WS2 ML), O-terminated nanoribbons (WS2 NR), OH-decorated WS2 ML, tungsten oxides, pristine/defective WS2 NR, pristine/defective WS2 ML, and finite WS2 clusters. Structural diversity is centred on functionalized basal planes and edges (in nanoribbon configurations) to resolve the energetics of $O_2$ and $H_2O$ interactions. The dataset includes a combination of bulk phases, surface defects, and finite clusters to ensure broad transferability across the $WS_2$–O–H potential energy surface.

To ensure high transferability, the dataset encompasses a broad range of coordination environments and stoichiometry (see Fig. S16). This includes reference pristine $WS_2$ supercells and various tungsten oxide ($WO_x$) phases sourced from the Materials Project. To resolve specific reactive pathways, over 70% of the dataset focuses on O-functionalized and OH-decorated basal planes and edges. This diversity was essential for accurately capturing oxidation kinetics and the influence of $H_2O$/OH interactions on various sites, including defects.

We incorporated 11 distinct types of basal plane defect configurations into the data set, and their rattled copies (Gaussian displacements of 0.05–0.15 Å). Additionally, finite $WS_2$ clusters and flakes were included to improve the model's performance in representing edge-terminations and small molecular fragments relevant to sublimation processes. The clusters were obtained from a data set made public by Hafizi et. al.[42]

**Adaptive Refinement and Landscape Exploration**

The dataset was expanded through an adaptive feedback loop, as the relevant reaction intermediates and sublimation products could not be fully identified a priori. The sampling progressed through the following stages:

MD Sampling: Initial configurations were generated via MD driven by the MACE-MP-0 foundational model (and subsequent models such as MACE-MP-02b) to explore thermal fluctuations.[16]

Path Sampling: Nudged Elastic Band (NEB) calculations were performed to sample specific assumed reaction coordinates for the initial phase. Configurations along the path were "fed back" into the training set over multiple refinement cycles (often 3 cycles). This feedback ensured the potential reached chemical accuracy in the relevant regions of the PES.

Global & Path Exploration: The data set was further expanded by including model-identified transition states and minima obtained via basin-hopping (GMIN) and discrete path sampling (OPTIM).[19,21] This approach was essential for reliably mapping reaction pathways.

**Model Training and Implementation**

The production models were developed by fine-tuning the large MACE-MP-02b model. We evaluated two transfer learning strategies: naïve fine-tuning (initialization from foundational weights) and a multi-head approach (replay of the MPTraj dataset).[43] As both methods yielded comparable accuracy, the final potential utilizes the naïve approach for its computational efficiency. While the general model encompasses a portion of the $WS_2$–O–H chemical space, a specialized hydrogen-free version was utilized for edge sublimation calculations to ensure maximum stability and accuracy in pathway searches. Both versions of the model are made available in the supplementary material.

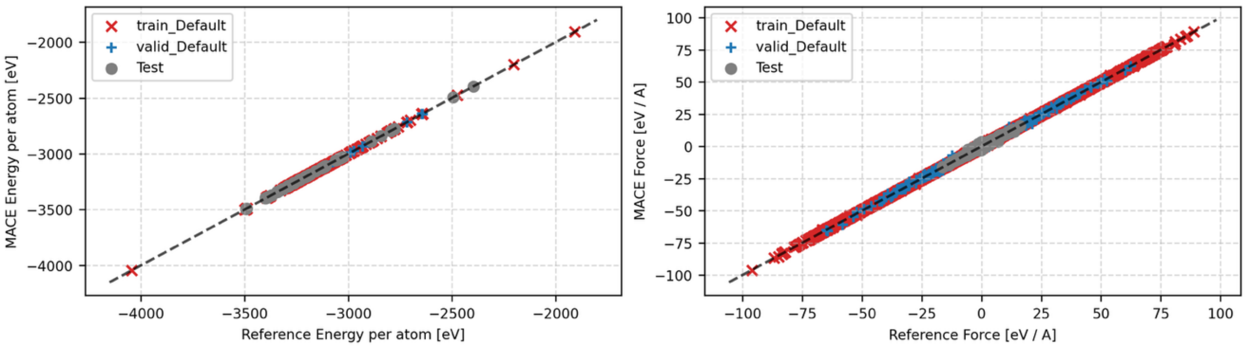


**Fig. S17 Regression performance of the MACE potential for the $WS_2$–O–H system.** Correlation plots for (left) total energy and (right) force components comparing DFT reference values against MLIP predictions for the independent test set (N = 339). The dashed diagonal line represents ideal parity. The model demonstrates high fidelity across the sampled chemical

space, achieving a Root Mean Square Error (RMSE) of 4.8 meV/atom for energies and 63.3 eV/Å for forces (2.38% relative force RMSE), indicating excellent agreement with the underlying electronic structure calculations.

### ii. Reaction Path Discovery and Barrier Calculations

To resolve the kinetic pathways of oxidation and sublimation, we employed a multi-tiered approach that balances the computational efficiency of the MACE potential with the high-level accuracy of Density Functional Theory (DFT).

#### Initial Screening and NEB Refinement

Reaction pathways were initially explored approximately using the Nudged Elastic Band (NEB) method as implemented in the ASE package.[14] By leveraging GPU acceleration, the MACE potential allowed for rapid exploration of the chemical space, providing a high-throughput first-approximation of transition states. Initial path searches were conducted with a force convergence criterion of $F_{max}$ = 0.1 eV/Å (maximum force component perpendicular to the path on any atom), subsequently refined to a tighter criterion of 0.05 eV/Å using the Climbing-Image NEB (CI-NEB) algorithm to approximately locate saddle points.[44]

#### DFT Validation of Elementary Steps

For elementary processes involving short reaction coordinates including sulfur removal at various surface sites and diffusion processes, the final MACE-generated paths were validated via single-point DFT evaluations. The accuracy of these paths was assessed by comparing the MACE energy profile to the DFT reference and examining the DFT force residuals, $F_{max}$. As illustrated in Fig. S19, the MACE potential demonstrates good agreement with DFT for these transitions.

#### Discrete Path Sampling for Multi-Step Processes

For all processes explored in this study, except the calculation in Fig. S18, paths connecting end states were evaluated with  Discrete Path Sampling (DPS) as implemented in the OPTIM.[45,46] This approach, based on accurate characterisation of transition states and connectivity, is better suited for landscapes containing multiple intermediate minima that are difficult to capture via standard NEB. For highly multi-step paths, such as edge sublimation, we utilized OPTIM directly. These simulations utilized a rigorous convergence framework to ensure the stability of the identified pathways:

- Local Minimization: Transitions and minima were converged using a customised LBFGS algorithm in GMIN and OPTIM with a gradient tolerance of $1.0 \times 10^{-6}$ eV/Å
- Energy Resolution: An energy difference tolerance ($E_{diff}$) of $1.0 \times 10^{-4}$ eV was maintained throughout the path connectivity search. This tolerance is combined with an aligned distance tolerance to identify identical stationary points.
- Step Control: Maximum displacement steps were constrained to 0.1 Å to maintain the integrity of the local atomic environments during LBFGS optimization.

Results reported for processes in the main text are derived from the OPTIM-based calculations, except for the sulfur removal diagram (Figure 4 in the main text). While some MACE-generated coordinates may exhibit minor force residuals when mapped to a DFT grid, the

topological agreement is sufficiently high that the overall kinetic barriers remain physically representative of the $WS_2$–O–H system.

### iii. Initial $O_2$ Chemisorption on various defects on $WS_2$

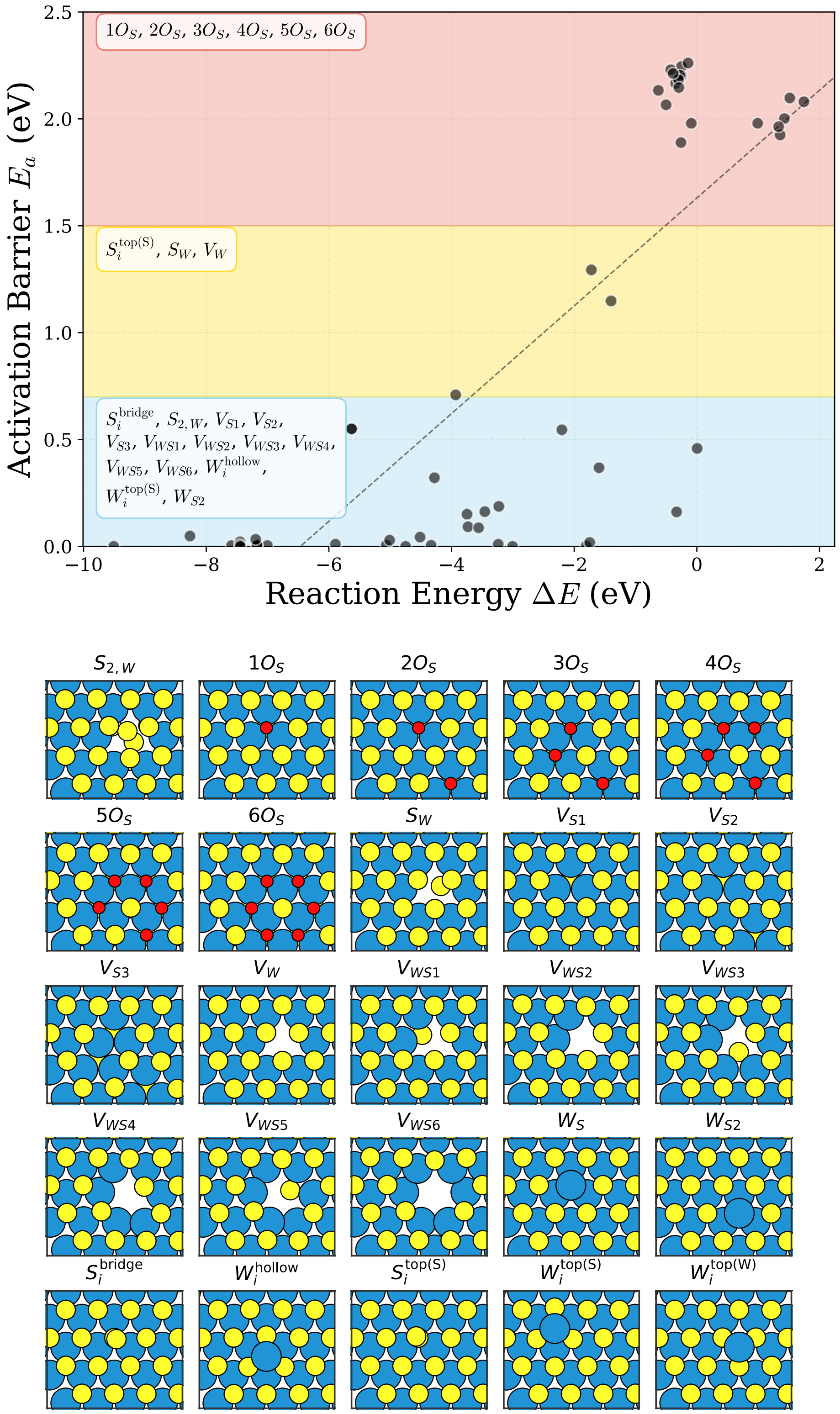


**Fig. S18 $O_2$ chemisorption reaction energetics for a wide variety of $WS_2$ defects.** (Top) Estimated activation energy ($E_a$) vs. reaction energy ($\Delta E$) for the most favourable $O_2$ adsorption pathways identified across a library of point defects and their structural permutations. Calculations used ASE's implementation of the NEB method and the trained MACE model. Defects are categorized into three kinetic regimes: highly active (blue, $E_a < 0.7$ eV), moderately

active (yellow, $0.7 \leq E_a \leq 1.5$ eV), and inactive (red, $E_a > 1.5$ eV). The lowest barrier per defect category and permutation is shown, representing the "best-case" reactivity for each site. (Bottom) Structural grid depicting the optimized geometries for each investigated defect. Blue, yellow, and red spheres denote W, S, and O atoms, respectively. For complex defects (e.g., $V_{S2}$), only one variant is shown for clarity.

We performed a large-scale screening of $O_2$ reactivity across a diverse library of point defects in the $WS_2$ basal plane. For each defect species, we explored multiple structural permutations, such as the relative positioning of vacancies in double sulfur vacancies ($V_{S3}$), to capture a greater variety of local environments. Reactivity was assessed by enumerating multiple start-end state pairs for each site, including $O_2$ dissociation across adjacent sulfur atoms, vacancy sites, and the formation of OSO–W intermediates. This culminated in hundreds of NEB calculations.

As shown in Fig. S18 (Top), the resulting activation barriers ($E_a$) are plotted against the reaction energies ($\Delta E$), revealing clear trends based on the local coordination environment. The defects were binned into three activity classes based on their $E_a$ relative to the thermal energy available at room temperature.

- Highly Active Class ($E_a < 0.7$ eV): This regime is dominated by defects with exposed or undercoordinated tungsten atoms (blue band), which provide strong electronic coupling for $O_2$ dissociation.
- Thermally Active Class (0.7–1.5 eV): Sites in this category typically feature undercoordinated sulfur atoms (yellow band), requiring moderate thermal activation to facilitate bond cleavage.
- Inactive Class ($E_a > 1.5$ eV): This region (red band) consists largely of pristine basal plane regions or substitutional oxygen ($O_S$) defects, where the lack of dangling bonds or significant surface strain leads to high kinetic barriers.

By reporting the lowest barrier found per defect permutation, we provide a lower bound for the onset of oxidation at specific defect centres, establishing a hierarchy of reactive sites for the initial stages of atmospheric degradation or thermal etching.

## iv. Initial Oxidation Near Various Sites on $WS_2$ for Sulfur Removal

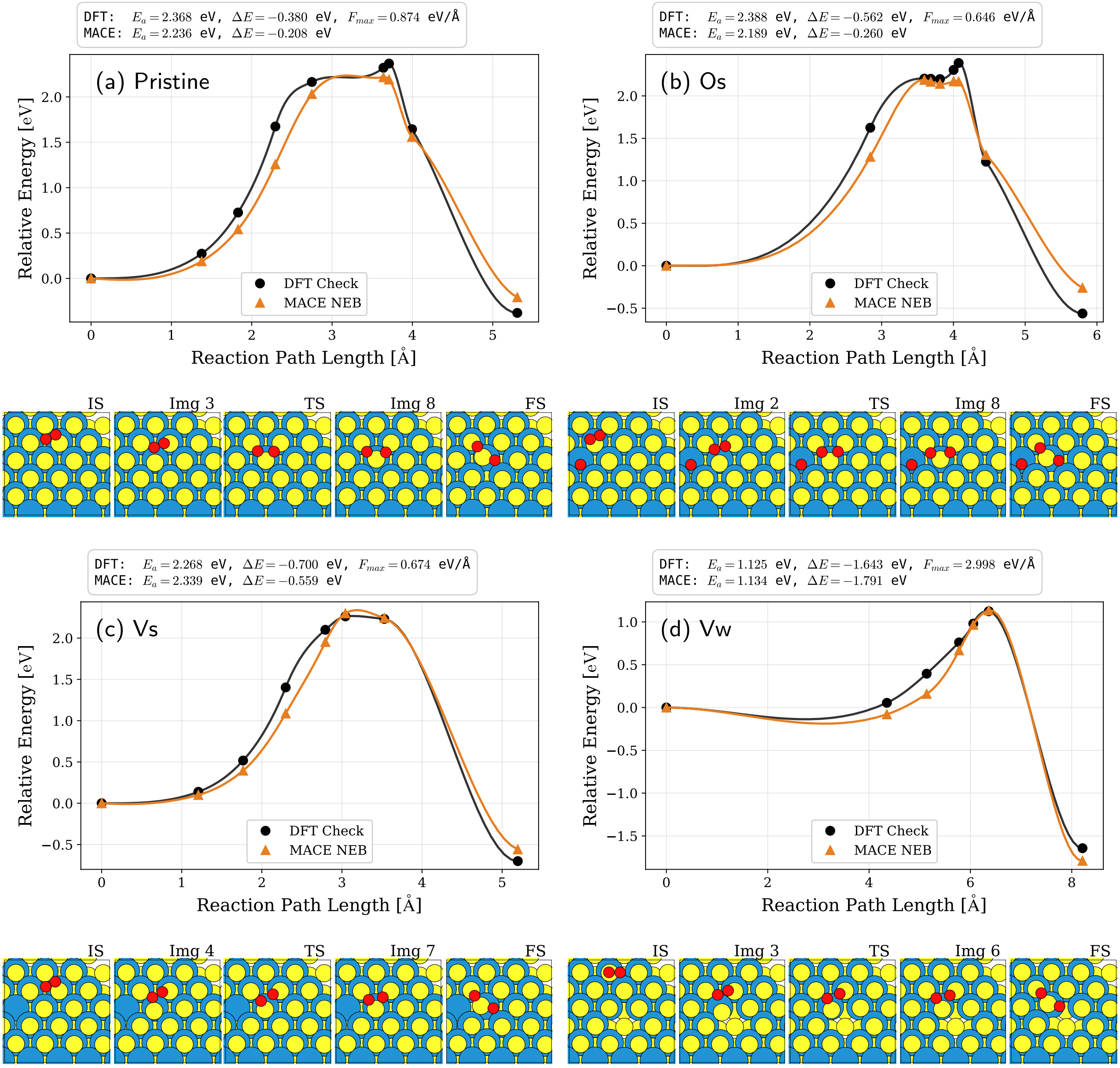


**Fig. S19 MACE potential validation and reaction manifold for $O_2$ chemisorption near basic defects on $WS_2$.** In each subfigure, (Top) Energy profile comparison between MACE NEB images (orange) and DFT (black) for approximate molecular oxygen dissociation and chemisorption paths. Annotated metrics indicate the corresponding estimated activation barrier ($E_a$), reaction energy ($\Delta E$), and maximum DFT force residual ($F_{max}$). (Bottom) Structural snapshots along the minimum energy path depicting the initial state (IS), an intermediate image/configuration, the highest image, which is the approximate transition state (TS) coinciding with O–O bond cleavage, another intermediate, and the final state (FS). Blue, yellow, and red spheres denote W, S, and O atoms, respectively.

### v. Sulfur Removal Pathway Computed using OPTIM

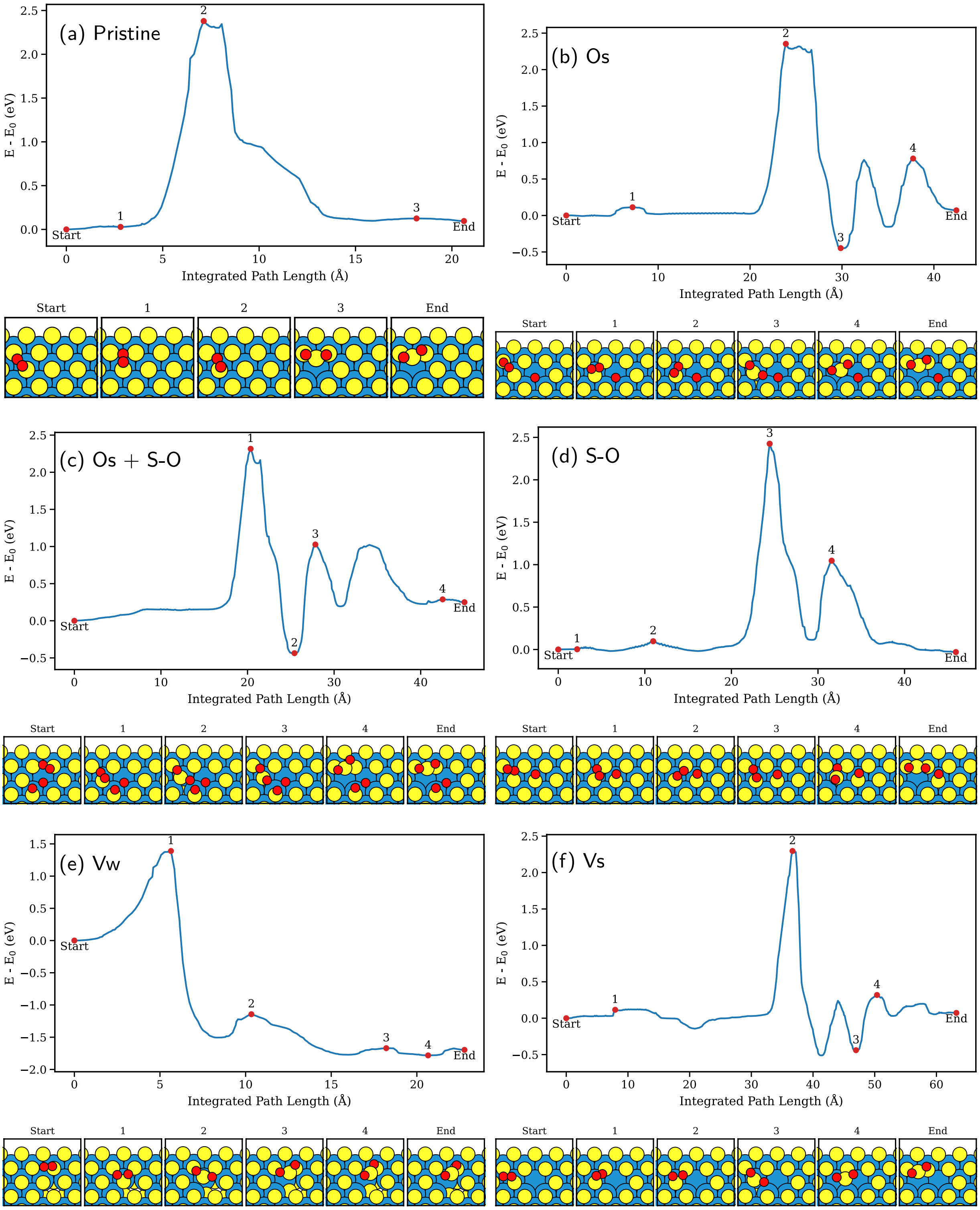


**Fig. S20 Oxygen-induced sulfur removal pathways (as $SO_2$) near basic defects as computed using OPTIM with MACE.** Sites explored include (a) the pristine basal plane, (b) substitutional oxygen ($O_S$), (c) an $O_S$ with a neighbouring oxygen adatom chemisorbed on top a sulfur atom (S-O) representing the oxidized/passivated form of a sulfur vacancy ($V_S$), (d) an S-O, and (e) a tungsten vacancy ($V_W$). Each subplot comprises the relative energy plotted

against the integrated path length (top) and snapshots of selected minima and transition states along the path (bottom). Blue, yellow, and red spheres denote W, S, and O atoms, respectively. Here we see that the correct paths are actually multistep, in contrast to the profiles corresponding to the ASE/NEB images.

### vi. Sulfur Removal Through SO Desorption

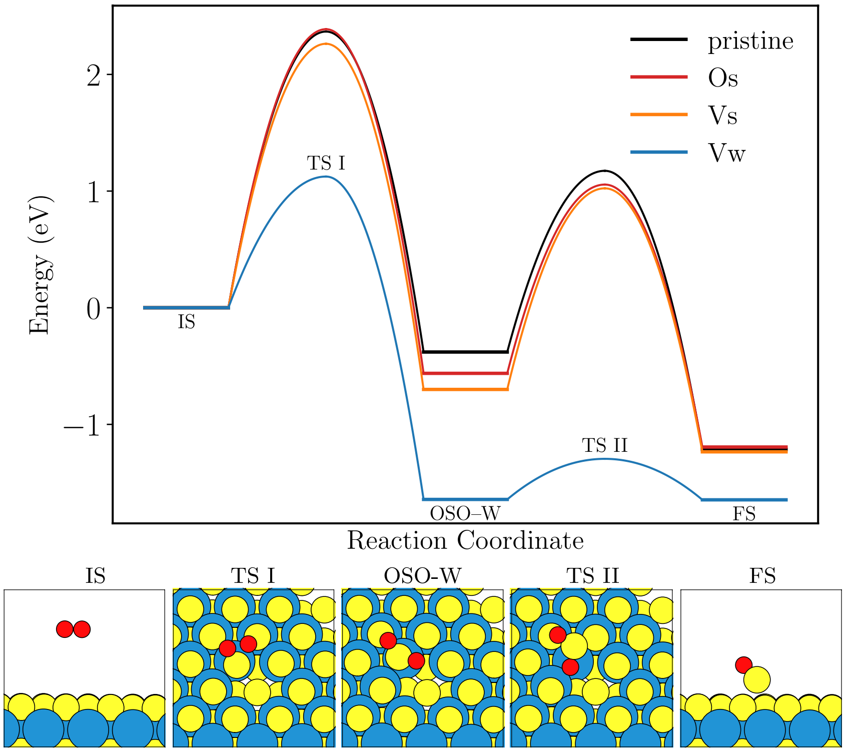


**Fig. S21 Oxygen-induced Sulfur removal as SO, near various basic defects as approximated by NEB using MACE:** (Top) Relative energies for the initial oxidation step near different sites including $O_2$ dissociation to form OSO-W followed by SO desorption leaving behind a substitutional oxygen. (Bottom) snapshots from the approximated NEB path showing the initial state (IS), two transition states (TS I and TS II), the important OSO-W intermediate and the final state (FS). Blue, yellow, and red spheres denote W, S, and O atoms, respectively. Sulfur monoxide (SO) release represents an alternative desorption product often associated with more prohibitive barriers than $SO_2$. Energies shown here are obtained by running DFT single-point calculations on selected configurations from the MACE-optimized NEB path.

### vii. Diffusion Pathways on $WS_2$ Basal Plane

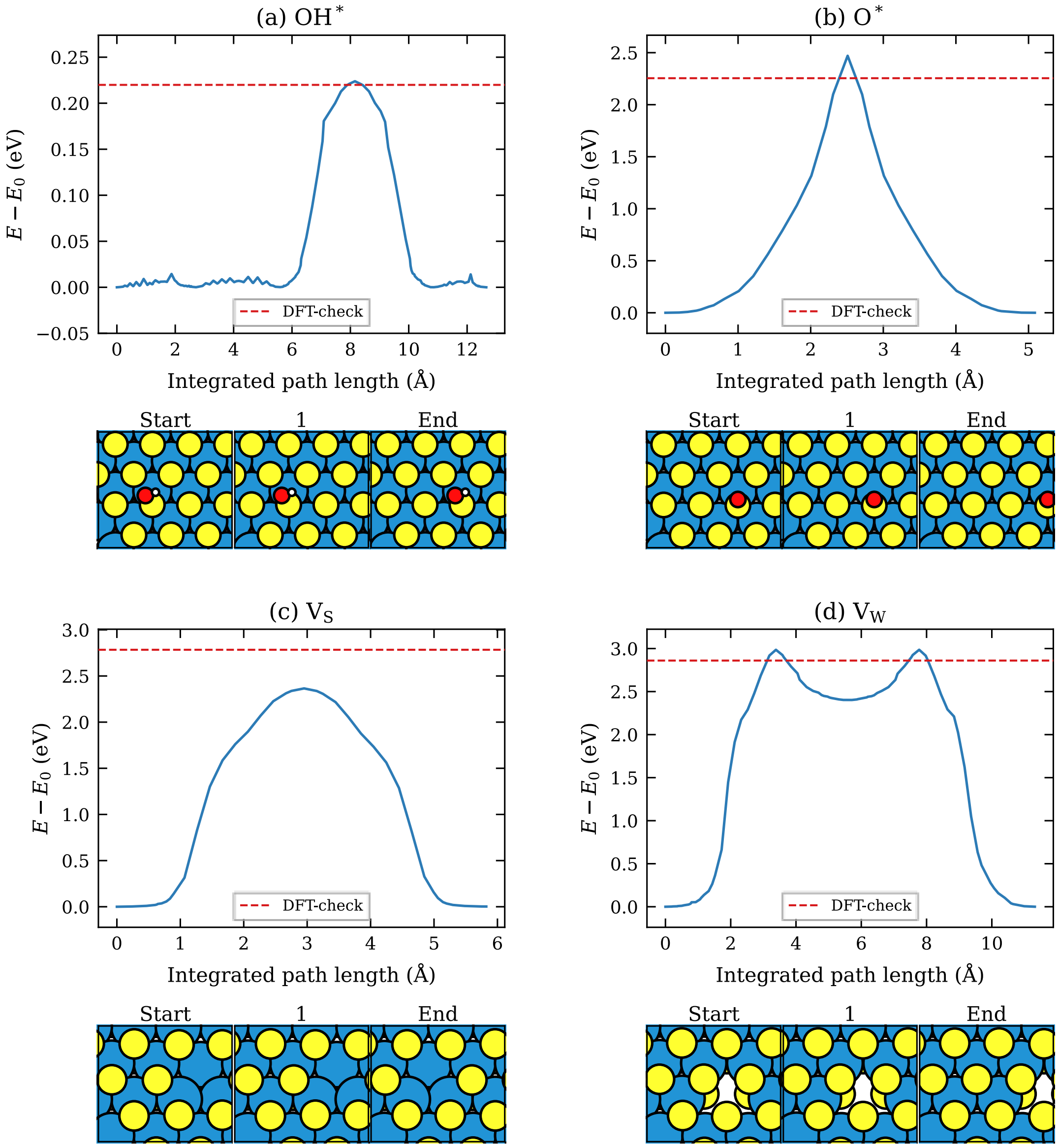


**Fig. S22 Diffusion pathways for four different candidates (defects and adsorbates) on $WS_2$ as computed using OPTIM with MACE.** Candidates include (a) chemisorbed hydroxyl ($OH^*$), (b) chemisorbed oxygen ($O^*$), (c) sulfur vacancy ($V_S$) and (d) a tungsten vacancy ($V_W$). Each subplot comprises the relative energy plotted against the integrated path length (top). DFT single point calculations were run for the initial, transition and end states. The red dashed lines show denote the difference in energy between the transition state and the initial state as computed with DFT. Snapshots of selected minima and transition states along the path are shown (bottom). Blue, yellow, red and white spheres denote W, S, O and H atoms, respectively. Water and molecular oxygen physisorb weakly to the surface and so were not considered.

Table S1: Diffusion barriers, coefficients and length scales for various species on the $WS_2$ basal plane

| Species | $E_D$ (eV) | D $\left(\frac{nm^2}{s}\right)$ | $\sqrt{Dt}$ (nm) |
|---|---|---|---|
| $O^*$ | 2.26 | $5 \times 10^{-5} - 10^{-6}$ | $7 \times 10^{-1} - 10^{-2}$ |
| $V_S$ | 2.78 | $1 \times 10^{-8} - 10^{-9}$ | $1 \times 10^{-3} - 3 \times 10^{-3}$ |
| $V_W$ | 2.86 | $3 \times 10^{-9} - 10^{-10}$ | $2 \times 10^{-3} - 5 \times 10^{-4}$ |
| $OH^*$ | 0.39 | $5 \times 10^{7} - 10^{8}$ | $2 \times 10^{5} - 7 \times 10^{5}$ |

Diffusion coefficients (D) were estimated using the Arrhenius expression: $D = D_0 \exp\left(\frac{-E_A}{k_B T}\right)$ where $D_0$ is the pre-exponential factor approximated by $D_0 = \frac{1}{4}\alpha^2 v_0$ Here, α is the lattice spacing (3.187Å) and $v_0$ is an attempt frequency which ranges between $10^{12}$ and $10^{13}$ $s^{-1}$ for most processes. From these coefficients, we make an additional simple estimate of the order of magnitude of the characteristic diffusion length scale by using $\sqrt{Dt}$ for a growth period of order 1000 s. The results are summarized in Table S1. The value of $E_D$ for $OH^*$ reported in Table S1 was derived following a DFT geometry optimization of the end states and then DFT NEB.

Among the candidates, $O_2$ was excluded due to its weak, room-temperature-level binding energy, which would not lead to a diffusion field. Likewise, commonly invoked mechanisms such as $O^*$ hopping, as well as $V_S$ and $V_W$ migration, proved too sluggish to account for the observed diffusion-limited growth. While sulfur vacancy migration can be accelerated under certain conditions (e.g., electron beam exposure or high defect concentrations), such scenarios are unlikely to explain the large-scale diffusion fields. We were unable to find an alternative, lower barrier, $O^*$ (subsurface) diffusion pathway as was reported in a study by Farigliano et. al on $MoS_2$.[47] In contrast, chemisorbed hydroxyl ($OH^*$) diffusion emerged as a plausible candidate, although substrate interactions and local chemical environments may significantly influence these diffusion barriers. Additionally, it should be noted that there is an error associated with DFT-PBE which may differ from experimental values. Consequently, further studies, including higher-level calculations and experimental validations, are necessary to conclusively identify the dominant diffusing species and refine our understanding of the underlying oxidation mechanisms in $WS_2$.

## viii. Initial $O_2$ reaction on W-ZZ and S-ZZ Edges Energy Profiles

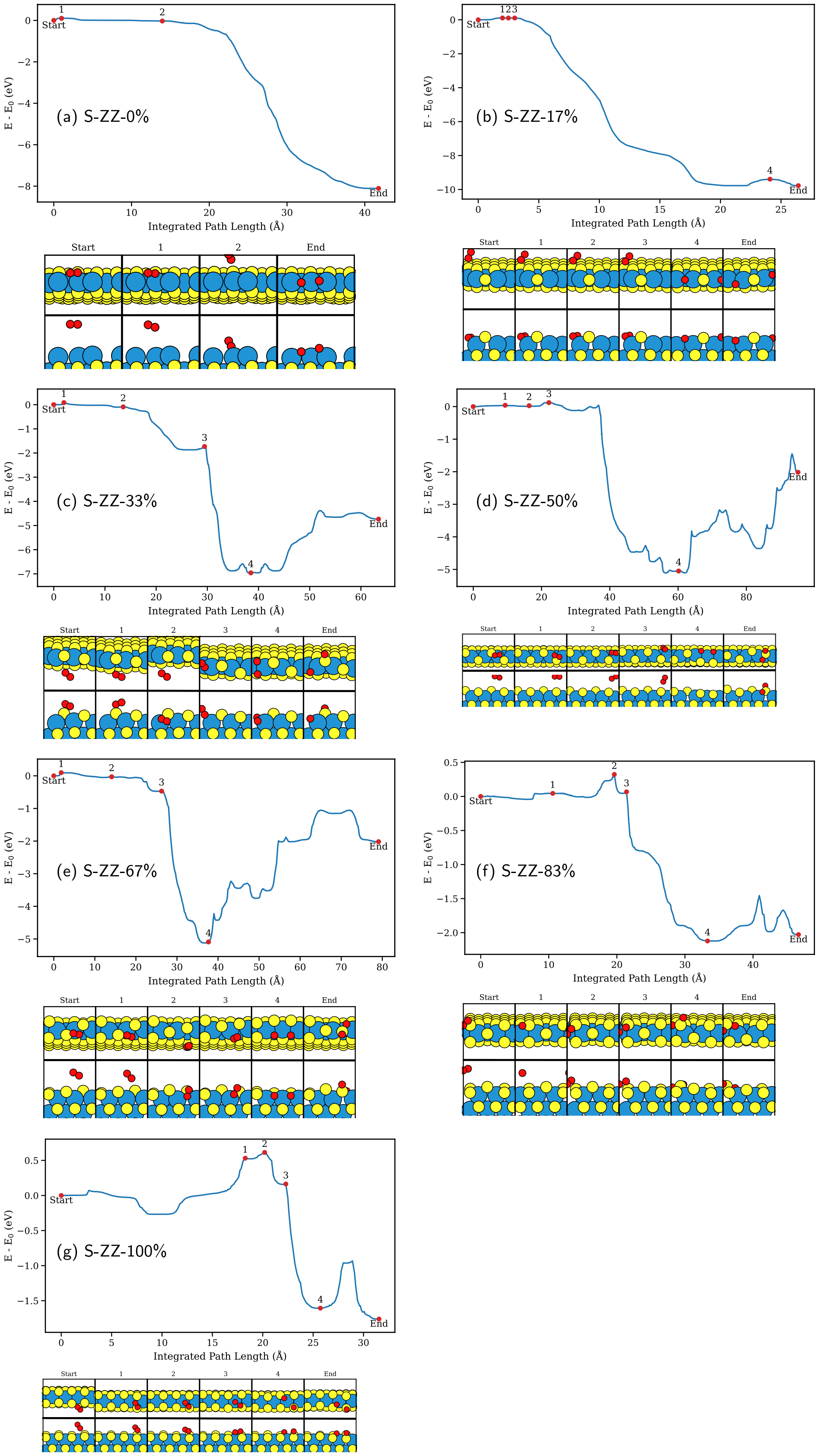

**Fig. S23 Pathways for the reactive adsorption of $O_2$ on the sulfur zigzag (S-ZZ) edges of various coverage computed using OPTIM with MACE.** Each subfigure is composed of the relative energy plotted against the integrated path length (top) and top/side views of selected snapshots along the reaction path (bottom). Snapshots selected typically comprise three high energy transition states and a relatively deep minimum. Labels (a-g) denote the coverage of the edge the profile/path correspond to. In several paths, the end state is not necessarily the lowest minimum along the path. For instance, in (e) intermediate 4 is a more stable product that is also more kinetically accessible.

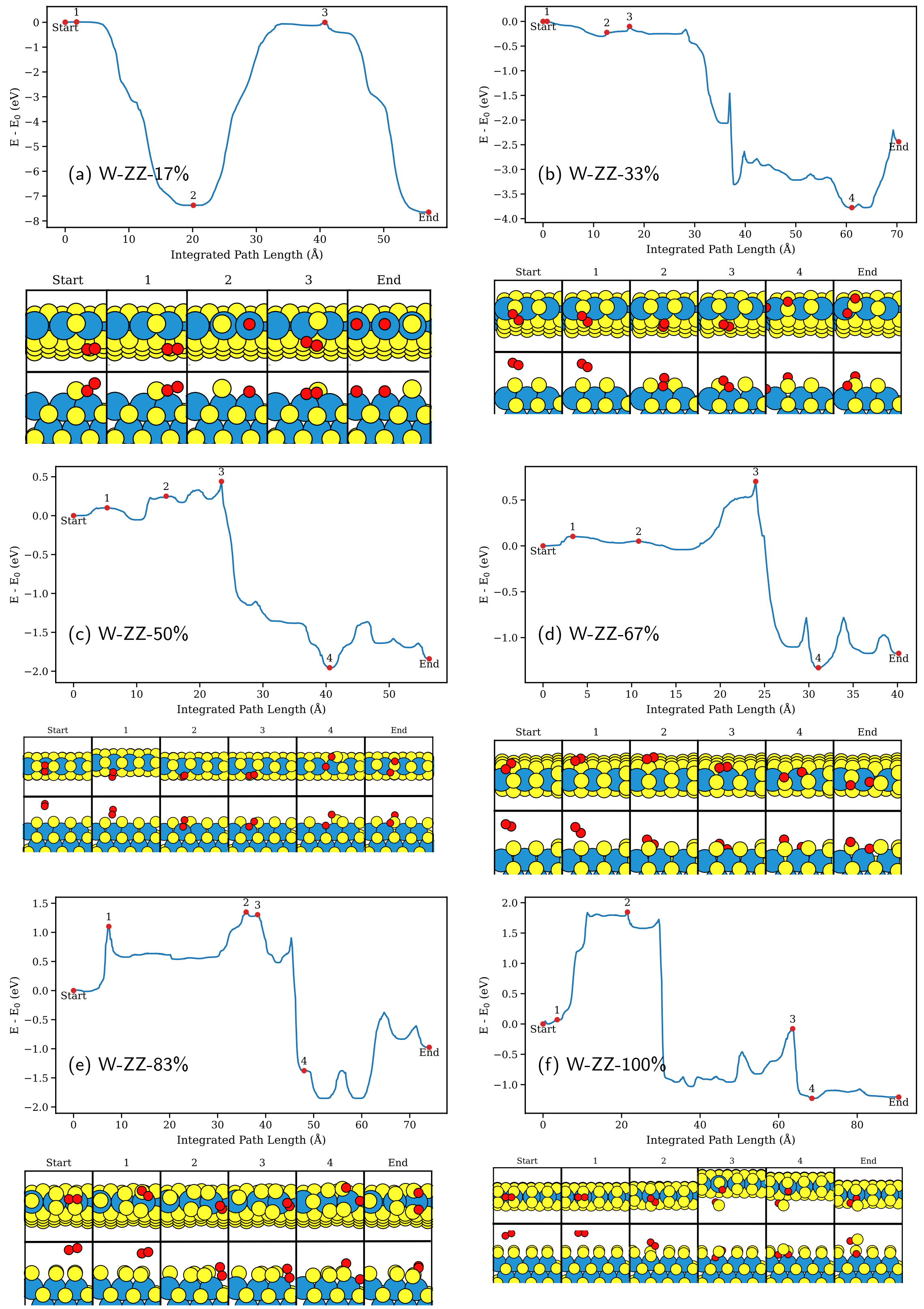


**Fig. S24 Pathways for the reactive adsorption of $O_2$ on the sulfur zigzag (S-ZZ) edges of various coverage computed using OPTIM with MACE.** Each subfigure is composed of the relative energy plotted against the integrated path length (top) and top/side views of selected snapshots along the reaction path (bottom). Snapshots selected typically comprise three high energy transition states and a relatively deep minimum. Labels (a-g) denote the coverage of

the edge the profile/path correspond to. In several paths, the end state is not necessarily the global minimum along the path. For instance, in (a) intermediate 2 is a more stable product that is also more kinetically accessible.

## XI. Supplementary Videos

**Supplementary Video S1: OSEM of oxidation of monolayer $WS_2$ at 680 °C**, related to Figures 1c/6a/7a in the manuscript.

**Supplementary Video S2: OSEM of oxidation of monolayer $WS_2$ at 450 °C**, related to Figures 3a/6b in the manuscript.

**Supplementary Video S3: Sulfur removal pathway near $V_W$ modelled using the MACE potential and discrete path sampling (OPTIM).** Atomistic simulation of the sulfur removal pathway in the vicinity of a tungsten vacancy ($V_W$), computed using the MACE ML potential and discrete path sampling as implemented in OPTIM. Atoms are coloured as follows: W (blue), S (yellow) and oxygen (red). The trajectory shows the minimum-energy pathway connecting initial and final states. Simulation frames are shown sequentially along the reaction coordinate. Related to Figure 4 in the main manuscript.

**Supplementary Video S4: Sublimation pathway of $WO_x$ species from the tungsten zigzag (W-ZZ) edge.** Atomistic simulation of the sublimation pathway of $WO_x$ species from the tungsten-terminated zigzag (W-ZZ) edge of monolayer $WS_2$. The pathway was computed using the MACE ML and discrete path sampling (OPTIM). W atoms are shown in blue, S in yellow, and O in red. Related to Figure 9b in the main manuscript.

**Supplementary Video S5: Sublimation pathway of $WO_x$ species from the sulfur zigzag (S-ZZ) edge.** Atomistic simulation of the sublimation pathway of $WO_x$ species from the sulfur-terminated zigzag (S-ZZ) edge of monolayer $WS_2$. The pathway was computed using the MACE ML potential and discrete path sampling (OPTIM). W atoms are shown in blue, S in yellow, and O in red. The trajectory represents the minimum-energy pathway between bound and desorbed configurations. Related to Figure 9a in the main manuscript.

## XII. References


(1) Rong, Y.; Fan, Y.; Leen Koh, A.; Robertson, A. W.; He, K.; Wang, S.; Tan, H.; Sinclair, R.; Warner, J. H. Controlling Sulphur Precursor Addition for Large Single Crystal Domains of WS2. *Nanoscale* **2014**, *6* (20), 12096–12103. https://doi.org/10.1039/C4NR04091K.

(2) Fan, Y.; Nakanishi, K.; Veigang-Radulescu, V. P.; Mizuta, R.; Stewart, J. C.; Swallow, J. E. N.; Dearle, A. E.; Burton, O. J.; Alexander-Webber, J. A.; Ferrer, P.; Held, G.; Brennan, B.; Pollard, A. J.; Weatherup, R. S.; Hofmann, S. Understanding Metal Organic Chemical Vapour Deposition of Monolayer WS2: The Enhancing Role of Au Substrate for Simple Organosulfur Precursors. *Nanoscale* **2020**, *12* (43), 22234–22244. https://doi.org/10.1039/D0NR06459A.

(3) Kotsakidis, J. C.; Zhang, Q.; Vazquez De Parga, A. L.; Currie, M.; Helmerson, K.; Gaskill, D. K.; Fuhrer, M. S. Oxidation of Monolayer WS2 in Ambient Is a Photoinduced Process. *Nano Lett.* **2019**, *19* (8), 5205–5215. https://doi.org/10.1021/ACS.NANOLETT.9B01599.

(4) Moelans, N.; Blanpain, B.; Wollants, P. An Introduction to Phase-Field Modeling of Microstructure Evolution. *Calphad* **2008**, *32* (2), 268–294. https://doi.org/10.1016/j.calphad.2007.11.003.

(5) Clark, S. J.; Segall, M. D.; Pickard, C. J.; Hasnip, P. J.; Probert, M. I. J.; Refson, K.; Payne, M. C. First Principles Methods Using CASTEP. *Zeitschrift fur Kristallographie* **2005**, *220* (5–6), 567–570. https://doi.org/10.1524/ZKRI.220.5.567.65075/XML.

(6) Perdew, J. P.; Burke, K.; Ernzerhof, M. Generalized Gradient Approximation Made Simple. *Phys. Rev. Lett.* **1996**, *77* (18), 3865. https://doi.org/10.1103/PhysRevLett.77.3865.

(7) Grimme, S.; Ehrlich, S.; Goerigk, L. Effect of the Damping Function in Dispersion Corrected Density Functional Theory. *J. Comput. Chem.* **2011**, *32* (7), 1456–1465. https://doi.org/10.1002/JCC.21759;JOURNAL:JOURNAL:1096987X;WGROUP:STRING:PUBLICATION.

(8) Hohenberg, P.; Kohn, W. Inhomogeneous Electron Gas. *Physical Review* **1964**, *136* (3B), B864. https://doi.org/10.1103/PhysRev.136.B864.

(9) Kohn, W.; Sham, L. J. Self-Consistent Equations Including Exchange and Correlation Effects. *Physical Review* **1965**, *140* (4A), A1133. https://doi.org/10.1103/PhysRev.140.A1133.

(10) Kieczka, D.; Durrant, T.; Milton, K.; Goh, K. E. J.; Bosman, M.; Shluger, A. Defects in WS2 Monolayer Calculated with a Nonlocal Functional: Any

Difference from GGA? *Electronic Structure* **2023**, *5* (2), 024001. https://doi.org/10.1088/2516-1075/ACC55D.

(11) Monkhorst, H. J.; Pack, J. D. Special Points for Brillouin-Zone Integrations. *Phys. Rev. B* **1976**, *13* (12), 5188. https://doi.org/10.1103/PhysRevB.13.5188.

(12) Payne, M. C.; Teter, M. P.; Allan, D. C.; Arias, T. A.; Joannopoulos, J. D. Iterative Minimization Techniques for Ab Initio Total-Energy Calculations: Molecular Dynamics and Conjugate Gradients. *Rev. Mod. Phys.* **1992**, *64* (4), 1045. https://doi.org/10.1103/RevModPhys.64.1045.

(13) Pulay, P. Convergence Acceleration of Iterative Sequences. the Case of Scf Iteration. *Chem. Phys. Lett.* **1980**, *73* (2), 393–398. https://doi.org/10.1016/0009-2614(80)80396-4.

(14) Hjorth Larsen, A.; JØrgen Mortensen, J.; Blomqvist, J.; Castelli, I. E.; Christensen, R.; Dułak, M.; Friis, J.; Groves, M. N.; Hammer, B.; Hargus, C.; Hermes, E. D.; Jennings, P. C.; Bjerre Jensen, P.; Kermode, J.; Kitchin, J. R.; Leonhard Kolsbjerg, E.; Kubal, J.; Kaasbjerg, K.; Lysgaard, S.; Bergmann Maronsson, J.; Maxson, T.; Olsen, T.; Pastewka, L.; Peterson, A.; Rostgaard, C.; SchiØtz, J.; Schütt, O.; Strange, M.; Thygesen, K. S.; Vegge, T.; Vilhelmsen, L.; Walter, M.; Zeng, Z.; Jacobsen, K. W. The Atomic Simulation Environment—a Python Library for Working with Atoms. *Journal of Physics: Condensed Matter* **2017**, *29* (27), 273002. https://doi.org/10.1088/1361-648X/AA680E.

(15) Gelžinytė, E.; Wengert, S.; Stenczel, T. K.; Heenen, H. H.; Reuter, K.; Csányi, G.; Bernstein, N. Wfl Python Toolkit for Creating Machine Learning Interatomic Potentials and Related Atomistic Simulation Workflows. *Journal of Chemical Physics* **2023**, *159* (12), 124801. https://doi.org/10.1063/5.0156845/2913657.

(16) Batatia, I.; Benner, P.; Chiang, Y.; Elena, A. M.; Kovács, D. P.; Riebesell, J.; Advincula, X. R.; Asta, M.; Avaylon, M.; Baldwin, W. J.; Berger, F.; Bernstein, N.; Bhowmik, A.; Bigi, F.; Blau, S. M.; Cărare, V.; Ceriotti, M.; Chong, S.; Darby, J. P.; De, S.; Della Pia, F.; Deringer, V. L.; Elijošius, R.; El-Machachi, Z.; Fako, E.; Falcioni, F.; Ferrari, A. C.; Gardner, J. L. A.; Gawkowski, M. J.; Genreith-Schriever, A.; George, J.; Goodall, R. E. A.; Grandel, J.; Grey, C. P.; Grigorev, P.; Han, S.; Handley, W.; Heenen, H. H.; Hermansson, K.; Ho, C. H.; Hofmann, S.; Holm, C.; Jaafar, J.; Jakob, K. S.; Jung, H.; Kapil, V.; Kaplan, A. D.; Karimitari, N.; Kermode, J. R.; Kourtis, P.; Kroupa, N.; Kullgren, J.; Kuner, M. C.; Kuryla, D.; Liepuoniute, G.; Lin, C.; Margraf, J. T.; Magdău, I. B.; Michaelides, A.; Moore, J. H.; Naik, A. A.; Niblett, S. P.; Norwood, S. W.; O'Neill, N.; Ortner, C.; Persson, K. A.; Reuter, K.; Rosen, A. S.; Rosset, L. A. M.; Schaaf, L. L.; Schran, C.; Shi, B. X.; Sivonxay, E.; Stenczel, T. K.; Sutton, C.; Svahn, V.; Swinburne, T. D.; Tilly, J.; van der Oord, C.; Vargas, S.; Varga-Umbrich, E.; Vegge, T.; Vondrák, M.; Wang, Y.; Witt, W. C.; Wolf, T.; Zills, F.; Csányi, G. A Foundation Model for Atomistic Materials Chemistry. *Journal*

*of Chemical Physics* **2025**, *163* (18), 184110. https://doi.org/10.1063/5.0297006/3372267.

(17) Batatia, I.; Kovacs, D. P.; Simm, G.; Ortner, C.; Csányi, G. MACE: Higher Order Equivariant Message Passing Neural Networks for Fast and Accurate Force Fields. *Adv. Neural Inf. Process. Syst.* **2022**, *35*, 11423–11436.

(18) Li, Z.; Scheraga, H. A. Monte Carlo-Minimization Approach to the Multiple-Minima Problem in Protein Folding. *Proceedings of the National Academy of Sciences* **1987**, *84* (19), 6611–6615. https://doi.org/10.1073/pnas.84.19.6611.

(19) Wales, D. J.; Doye, J. P. K. Global Optimization by Basin-Hopping and the Lowest Energy Structures of Lennard-Jones Clusters Containing up to 110 Atoms. *J. Phys. Chem. A* **1997**, *101* (28), 5111–5116. https://doi.org/10.1021/jp970984n.

(20) Wales, D. J.; Scheraga, H. A. Global Optimization of Clusters, Crystals, and Biomolecules. *Science (1979).* **1999**, *285* (5432), 1368–1372. https://doi.org/10.1126/science.285.5432.1368.

(21) WALES, D. J. Discrete Path Sampling. *Mol. Phys.* **2002**, *100* (20), 3285–3305. https://doi.org/10.1080/00268970210162691.

(22) Wales *, D. J. Some Further Applications of Discrete Path Sampling to Cluster Isomerization. *Mol. Phys.* **2004**, *102* (9–10), 891–908. https://doi.org/10.1080/00268970410001703363.

(23) Wales, D. J. Exploring Energy Landscapes. *Annu. Rev. Phys. Chem.* **2018**, *69* (1), 401–425. https://doi.org/10.1146/annurev-physchem-050317-021219.

(24) Trygubenko, S. A.; Wales, D. J. A Doubly Nudged Elastic Band Method for Finding Transition States. *J. Chem. Phys.* **2004**, *120* (5), 2082–2094. https://doi.org/10.1063/1.1636455.

(25) Sheppard, D.; Terrell, R.; Henkelman, G. Optimization Methods for Finding Minimum Energy Paths. *Journal of Chemical Physics* **2008**, *128* (13), 134106. https://doi.org/10.1063/1.2841941/977389.

(26) Mills, G.; Jónsson, H.; Schenter, G. K. Reversible Work Transition State Theory: Application to Dissociative Adsorption of Hydrogen. *Surf. Sci.* **1995**, *324* (2–3), 305–337. https://doi.org/10.1016/0039-6028(94)00731-4.

(27) Munro, L. J.; Wales, D. J. Defect Migration in Crystalline Silicon. *Phys. Rev. B* **1999**, *59* (6), 3969. https://doi.org/10.1103/PhysRevB.59.3969.

(28) Henkelman, G.; Jónsson, H. A Dimer Method for Finding Saddle Points on High Dimensional Potential Surfaces Using Only First Derivatives. *J. Chem. Phys.* **1999**, *111* (15), 7010–7022. https://doi.org/10.1063/1.480097.

(29) Kumeda, Y.; Wales, D. J.; Munro, L. J. Transition States and Rearrangement Mechanisms from Hybrid Eigenvector-Following and Density Functional Theory.: Application to C10H10 and Defect Migration in Crystalline Silicon.

*Chem. Phys. Lett.* **2001**, *341* (1–2), 185–194. https://doi.org/10.1016/S0009-2614(01)00334-7.

(30) Carr, J. M.; Trygubenko, S. A.; Wales, D. J. Finding Pathways between Distant Local Minima. *Journal of Chemical Physics* **2005**, *122* (23), 37. https://doi.org/10.1063/1.1931587/901064.

(31) Griffiths, M.; Niblett, S. P.; Wales, D. J. Optimal Alignment of Structures for Finite and Periodic Systems. *J. Chem. Theory Comput.* **2017**, *13* (10), 4914–4931. https://doi.org/10.1021/ACS.JCTC.7B00543.

(32) Wales, D. J.; Carr, J. M. Quasi-Continuous Interpolation Scheme for Pathways between Distant Configurations. *J. Chem. Theory Comput.* **2012**, *8* (12), 5020–5034. https://doi.org/10.1021/CT3004832.

(33) Wales, D. J.; Head-Gordon, T. Evolution of the Potential Energy Landscape with Static Pulling Force for Two Model Proteins. *Journal of Physical Chemistry B* **2012**, *116* (29), 8394–8411. https://doi.org/10.1021/JP211806Z.

(34) Rowe, J.; Röder, K. Chemical Bonds in Collagen Rupture Selectively under Tensile Stress. *Physical Chemistry Chemical Physics* **2023**, *25* (3), 2331–2341. https://doi.org/10.1039/D2CP05051J.

(35) Toth, M.; Lobo, C.; Friedli, V.; Szkudlarek, A.; Utke, I. Continuum Models of Focused Electron Beam Induced Processing. *Beilstein Journal of Nanotechnology* **2015**, *6* (1), 1518–1540. https://doi.org/10.3762/bjnano.6.157.

(36) Sanz-Hernández, D.; Fernández-Pacheco, A. Modelling Focused Electron Beam Induced Deposition beyond Langmuir Adsorption. *Beilstein Journal of Nanotechnology* **2017**, *8* (1), 2151–2161. https://doi.org/10.3762/bjnano.8.214.

(37) Frank, F. C. On the Kinematic Theory of Crystal Growth and Dissolution Processes, II. *Zeitschrift fur Physikalische Chemie* **1972**, *77* (1–6), 84–92. https://doi.org/10.1524/ZPCH.1972.77.1-6.084/XML.

(38) Morgan, D. J. Core-Level Spectra of Powdered Tungsten Disulfide, WS2. *Surface Science Spectra* **2018**, *25* (1). https://doi.org/10.1116/1.5030093.

(39) Daniel, M. F.; Desbat, B.; Lassegues, J. C.; Gerand, B.; Figlarz, M. Infrared and Raman Study of WO3 Tungsten Trioxides and WO3, XH2O Tungsten Trioxide Tydrates. *J. Solid State Chem.* **1987**, *67* (2), 235–247. https://doi.org/10.1016/0022-4596(87)90359-8.

(40) Li, H.; Lu, G.; Wang, Y.; Yin, Z.; Cong, C.; He, Q.; Wang, L.; Ding, F.; Yu, T.; Zhang, H. Mechanical Exfoliation and Characterization of Single- and Few-Layer Nanosheets of WSe2, TaS2, and TaSe2. *Small* **2013**, *9* (11), 1974–1981. https://doi.org/10.1002/smll.201202919.

(41) Yamamoto, M.; Dutta, S.; Aikawa, S.; Nakaharai, S.; Wakabayashi, K.; S. Fuhrer, M.; Ueno, K.; Tsukagoshi, K. Self-Limiting Layer-by-Layer Oxidation of Atomically Thin WSe2. *Nano Lett.* **2015**, *15* (3), 2067–2073. https://doi.org/10.1021/nl5049753.

(42) Hafizi, R.; Ghasemi, S. A.; Hashemifar, S. J.; Akbarzadeh, H. A Neural-Network Potential through Charge Equilibration for WS2: From Clusters to Sheets. *Journal of Chemical Physics* **2017**, *147* (23), 234306. https://doi.org/10.1063/1.5003904/195571.

(43) Batatia, I.; Lin, C.; Hart, J.; Kasoar, E.; Elena, A. M.; Norwood, S. W.; Wolf, T.; Csányi, G. Cross Learning between Electronic Structure Theories for Unifying Molecular, Surface, and Inorganic Crystal Foundation Force Fields. **2025**.

(44) Henkelman, G.; Uberuaga, B. P.; Jónsson, H. A Climbing Image Nudged Elastic Band Method for Finding Saddle Points and Minimum Energy Paths. *J. Chem. Phys.* **2000**, *113* (22), 9901–9904. https://doi.org/10.1063/1.1329672.

(45) WALES, D. J. Discrete Path Sampling. *Mol. Phys.* **2002**, *100* (20), 3285–3305. https://doi.org/10.1080/00268970210162691.

(46) Wales, D. J. Some Further Applications of Discrete Path Sampling to Cluster Isomerization. *Mol. Phys.* **2004**, *102* (9–10), 891–908. https://doi.org/10.1080/00268970410001703363.

(47) Farigliano, L. M.; Paredes-Olivera, P. A.; Patrito, E. M. Initial Steps of Oxidative Etching of MoS2 Basal Plane Induced by O2. *The Journal of Physical Chemistry C* **2020**, *124* (24), 13177–13186. https://doi.org/10.1021/ACS.JPCC.0C02141.
